# Trends in Human Papillomavirus–Associated Cancers, Demographic Characteristics, and Vaccinations in the US, 2001-2017

**DOI:** 10.1001/jamanetworkopen.2022.2530

**Published:** 2022-03-16

**Authors:** Cheng-I. Liao, Alex Andrea Francoeur, Daniel S. Kapp, Michelle Ann P. Caesar, Warner K. Huh, John K. Chan

**Affiliations:** 1 Department of Obstetrics and Gynecology, Kaohsiung Veterans General Hospital, Kaohsiung, Taiwan; 2 Department of Obstetrics and Gynecology, University of California, Los Angeles; 3 Department of Radiation Oncology, Stanford University School of Medicine, Stanford, California; 4 California Pacific/Palo Alto Medical Foundation/Sutter Research Institute, San Francisco, California; 5 Department of Obstetrics and Gynecology, University of Alabama Birmingham, Birmingham; 6 Division of Gynecologic Oncology, California Pacific/Palo Alto/Sutter Health Research Institute, San Francisco

## Abstract

**Importance:**

Nearly 45 000 human papillomavirus (HPV)-associated cancers are diagnosed annually in the US. The HPV vaccine has been approved since 2006, but information on the association between vaccination and the incidence of HPV-attributable cancers is unclear.

**Objective:**

To evaluate the potential association of screening and vaccination on the trends of HPV-associated cancers.

**Design, Setting, and Participants:**

A retrospective, population-based cross-sectional study was conducted using data on HPV-associated (oropharyngeal squamous cell carcinoma [SCC], anal/rectal SCC, vulvar SCC, vaginal SCC, cervical carcinoma, and penile SCC) cancers from the US Cancer Statistics Public Use Database, representing 99% of the US population, between January 1, 2001, and December 31, 2017; HPV vaccination and screening data from the Behavioral Risk Factor Surveillance between January 1, 2001, and December 31, 2016; and TeenVaxView between January 1, 2008, and December 31, 2018. National Cancer Database and Behavioral Risk Factor Surveillance were used to correct for hysterectomy. Data analysis was performed from April 1, 2020, to June 30, 2021.

**Exposures:**

Patient demographic characteristics, including age, race and ethnicity, sex, region, and vaccination status.

**Main Outcomes and Measures:**

The main outcomes examined in this study were diagnoses of any HPV-associated cancer and HPV vaccination status.

**Results:**

A total of 657 317 HPV-associated cancers (exact ages not collected by the United States Cancer Statistics); of these, 264 019 (40.2%) developed in men and 393 298 (59.8%) in women; 14 520 individuals (2.2%) were non-Hispanic Asian/Pacific Islander, 74 641 (11.4%) were non-Hispanic Black, 59 841 (9.1%) were Hispanic, and 499 899 were non-Hispanic White (76.1%). More than half (206 075 [52.4%]) of cancers in women were cervical, whereas most (211 421 [80.1%]) cancers in men were oropharyngeal. In female adolescents (aged 13-17 years), the vaccination rate increased from 37.2% to 69.9% from 2008 to 2018 (annual percent change: 6.57% [95% CI, 5.83%-7.32%]). Before vaccination approval, cervical cancer rates in the 20- to 24-year age group were decreasing at 2.29% annually (*P* = .045); after vaccine approval, this rate has been decreasing at 9.50% (*P* = .003). In men, annual increases were noted in oropharyngeal (2.71%) and anal/rectal (1.83%) cancers (*P* < .001); in women, the incidence of oropharyngeal remained stable but anal/rectal cancer increased at 2.83% every year (*P* < .001).

**Conclusions and Relevance:**

In the US, cervical cancer rates have decreased at a population level, especially in younger women. The findings of this study suggest this decrease may be associated with vaccination. Given the increase in oropharyngeal and anal/rectal cancers, particularly in men, it may be important to highlight vaccination uptake in both sexes.

## Key Points

**Question:**

Are human papillomavirus (HPV) vaccinations and screening associated with trends in HPV-associated cancers in the US?

**Findings:**

In this cross-sectional study using 3 national databases, the incidence of cervical cancer has decreased, especially in younger women, after HPV vaccine approval. However, oropharyngeal and anal/rectal cancers have increased among men.

**Meaning:**

These findings suggest that the decrease in the incidence of cervical cancers, particularly in young women may be associated with HPV vaccination approval; however, it may be too early to evaluate this association in oropharyngeal and anal cancers, which occur later in life.

## Teaser

This cross-sectional study examines changes in the incidence of human papillomavirus–associated cancers in the US and the rate of screening and vaccination before and after approval of the vaccine.

## Introduction

Approximately 45 000 human papillomavirus (HPV)-associated cancers are diagnosed annually in the US, with nearly 60% detected in women and 40% in men.^1,2,3^ Many cervical, anal, vaginal, oropharyngeal, vulvar, and penile cancers are attributable to HPV.^4^ Although cervical cancers are mostly preventable with screening, other HPV-associated cancers do not have any screening guidelines.^1,5^ The HPV vaccine was approved by the US Food and Drug Administration in 2006 for women and 2009 for men, providing protection against 90% of cervical cancer–causing strains of HPV.^6^ Currently in the US, the HPV vaccine is recommended for males and females ages 9 to 26 years and can be offered to selected individuals aged 27 to 45 years with shared clinical decision-making. The vaccine is also approved for prevention of anal, vulvar, and oropharyngeal cancers in men and women.

Although many studies have examined the incidence of invasive cervical cancer and its association with HPV, few have examined the trends of all HPV-associated cancers across both sexes.^3,7^ This cross-sectional study also evaluated HPV-associated cancers that can vs cannot be identified through screening to determine the potential association of vaccination with the incidence of these cancers. Using a large, national database representing approximately 99% of the population of the US, we sought to evaluate the trends in HPV-associated cancer incidence by age, race and ethnicity, and tumor stage from 2001 to 2017.

## Methods

### Data Sources

Deidentified cancer incidence data are reported to the Centers for Disease Control and Prevention National Program of Cancer Registries and the National Cancer Institute Surveillance, Epidemiology, and End Results (SEER) Program. Cancer surveillance data from the Centers for Disease Control and Prevention and National Cancer Institute are combined to become United States Cancer Statistics (USCS), the official source for federal cancer data. Incidences and trends were calculated using the USCS public use databases including cancer incidence and population data for all 50 states and the District of Columbia. These data provide information on more than 31 million cancer cases, representing 99% of the US population.^8^ The 2019 USCS data submission (2001-2017) was used for analysis.^9^ As the largest health survey system in the world, the Behavioral Risk Factor Surveillance System (BRFSS) conducts more than 400 000 adult interviews per year.^10^ Data from the BRFSS survey from 2001 to 2016 were used to determine the trends of cervical cytologic screening (female, age >18 years), rate of HPV vaccination (male and female, age 18-44 years), and hysterectomy (female, age 18-44 years).

Teen vaccination coverage data are collected through the National Immunization Survey–Teen. The National Immunization Survey–Teen is a random-digit dialed survey of parents or guardians of adolescents aged 13 to 17 years and, in 2018, included more than 20 000 adolescents. The survey is followed by a questionnaire mailed to vaccination providers to obtain vaccination history.^11^ TeenVaxView data in 50 states and the District of Columbia from 2008 to 2018 were used in this data analysis.

The nationally recognized National Cancer Database is sourced from hospital registry data that are collected in more than 1500 Commission on Cancer–accredited facilities. Data represent more than 70% of newly diagnosed cancer cases nationwide and more than 34 million historical records. The National Cancer Database and BRFSS were used to correct for hysterectomies in order to calculate cervical cancer incidence as previously described.^12^

Because all data were obtained from published data, this study does not constitute human subjects research and does not require institutional review board review or exemption according to the US Department of Health and Human Services (45 CFR §46). This study followed the Strengthening the Reporting of Observational Studies in Epidemiology (STROBE) reporting guideline for cross-sectional studies.

### Variables

Cancers associated with HPV were confirmed by microscopy and classified by the Centers for Disease Control and Prevention as oropharyngeal squamous cell carcinoma (SCC), anal/rectal SCC, vulvar SCC, vaginal SCC, penile SCC, and cervical carcinoma^13^ (eTable 1 in the Supplement). In cervical cancer, histologic subtypes were divided into carcinoma (including SCC, adenocarcinoma, adenosquamous carcinoma, and other carcinoma) and noncarcinoma according to SEER Cancer Statistics Review 1975-2018 by the *International Classification of Diseases for Oncology, Third Edition* codes^14^ (eTable 2 in the Supplement). Self-reported race and ethnicity included non-Hispanic Asian or Pacific Islander (Asian/Pacific Islander hereafter), non-Hispanic Black (Black hereafter), Hispanic, non-Hispanic White (White hereafter), and other/unknown according to the SEER algorithm.^15^ Disparities in incidence rates based on race and ethnicity in HPV-associated cancers have been previously reported.^3^

According to the US Census Bureau, the regions of the US were divided into Northeast, South, Midwest, and West. Stage of cancer was categorized as local, regional, distant, and unknown.^16^ Regional stage is defined as spread to local lymph nodes; distant disease is defined as metastatic to surrounding organs or distant structures. These data sets were divided into age groups at 5-year intervals.

### Statistical Analysis

SEER*Stat software, version 8.3.8 (released September 2, 2020; National Cancer Institute) was used to abstract the relevant data of USCS by cancer type, histologic type, race and ethnicity, region, stage, and age group. SPSS Statistics, version 27 (IBM Corp) was used to abstract the relevant data of BRFSS (by race and ethnicity, age group, cytologic smear, and HPV vaccination), TeenVaxView (by race and ethnicity and sex), and National Cancer Database. Joinpoint Regression Program, version 4.8.0.1 (National Cancer Institute) was used to calculate trends. In the study period, the incidence may change direction. The changing point is the joinpoint and connects the segment of incidence. The linear segment on a log scale changes with a constant annual percent change (APC); APC can be compared because it is a relative metric. The Grid Search method was selected and the maximum number of joinpoints was set at 3.^17^ Age-adjusted incidence rates were calculated per 100 000 persons and standardized to the 2000 US population. Age-specific incidence rates were crude rates by age group. Trends are described using APC with 95% CIs in each segment and average annual percent change (AAPC) with 95% CIs in the whole interval. The AAPC is derived by first estimating the underlying joinpoint model that best fits the data. The AAPC over any fixed interval is calculated using a weighted average of the slope coefficients of the underlying joinpoint regression line with the weights equal to the length of each segment over the interval. The final step of the calculation transforms the weighted average of slope coefficients to an APC.

## Results

### Patient Characteristics

Using the US Cancer Registry, a total of 657 317 HPV-associated cancers were identified from 2001 to 2017 (Table 1). Of those, 393 298 occurred in women (59.8%) and 264 019 occurred in men (40.2%). More than half (206 075 [52.4%]) of cancers in women were cervical, whereas most (211 421 [80.1%]) cancers in men were oropharyngeal. Based on race and ethnicity, 14 520 (2.2%) individuals were Asian/Pacific Islander, 74 641 (11.4%) were Black, 59 841 (9.1%) were Hispanic, and 499 899 (76.1%) were White. The USCS only provides the age group of the patients. It does not provide the exact age of patients; thus, we cannot calculate the mean or median age. The median age group was 50 to 54 years. Further information on baseline patient characteristics is reported in Table 1.

**Table 1.** Demographic and Clinical Characteristics of HPV-Associated Cancers in United States Cancer Statistics Public Use Database From 2001 to 2017

Characteristic	Male vs female, No. (%)^a^										
	Total	HPV-associated cancers		Oropharyngeal SCC		Anal/rectal SCC		Penile SCC	Vulvar SCC	Vaginal SCC	Cervical carcinoma
		Male	Female	Male	Female	Male	Female	Male	Female	Female	Female
Cases	657 317	264 019 (40.2)	393 298 (59.8)	211 421 (32.2)	51 400 (7.8)	32 679 (5.0)	62 721 (9.5)	19 919 (3.0)	59 559 (9.1)	13 543 (2.0)	206 075 (31.4)
Age group, y											
0-19	239 (0.0)	29 (0.0)	210 (0.1)	20 (0.0)	0	0	0	0	0	0	185 (0.1)
20-24	2276 (0.3)	122 (0.0)	2154 (0.5)	62 (0.0)	55 (0.1)	41 (0.1)	NA	19 (0.1)	72 (0.1)	0	2011 (1.0)
25-29	10 063 (1.5)	376 (0.1)	9687 (2.5)	170 (0.1)	114 (0.2)	145 (0.4)	84 (0.1)	61 (0.3)	254 (0.4)	34 (0.3)	9201 (4.5)
30-34	21 186 (3.2)	1166 (0.4)	20 020 (5.1)	520 (0.2)	301 (0.6)	444 (1.4)	318 (0.5)	202 (1.0)	784 (1.3)	104 (0.8)	18 513 (9.0)
35-39	31 287 (4.8)	3551 (1.3)	27 736 (7.1)	2019 (1.0)	696 (1.4)	1183 (3.6)	1025 (1.6)	349 (1.8)	1619 (2.7)	238 (1.8)	24 158 (11.7)
40-44	45 935 (7.0)	10 558 (4.0)	35 377 (9.0)	7338 (3.5)	1675 (3.3)	2502 (7.7)	2755 (4.4)	718 (3.6)	2971 (5.0)	529 (3.9)	27 447 (13.3)
45-49	64 144 (9.8)	23 823 (9.0)	40 321 (10.3)	18 921 (8.9)	3790 (7.4)	3865 (11.8)	5540 (8.8)	1037 (5.2)	4576 (7.7)	788 (5.8)	25 627 (12.4)
50-54	83 719 (12.7)	39 128 (14.8)	44 591 (11.3)	33 050 (15.6)	6448 (12.5)	4664 (14.3)	8686 (13.8)	1414 (7.1)	5640 (9.5)	1097 (8.1)	22 720 (11.0)
55-59	93 067 (14.2)	47 832 (18.1)	45 235 (11.5)	41 228 (19.5)	7984 (15.5)	4746 (14.5)	9833 (15.7)	1858 (9.3)	5765 (9.7)	1418 (10.5)	20 235 (9.8)
60-64	85 622 (13.0)	44 878 (17.0)	40 744 (10.4)	38 371 (18.1)	7887 (15.3)	4138 (12.7)	9186 (14.6)	2369 (11.9)	5823 (9.8)	1415 (10.4)	16 433 (8.0)
65-69	71 371 (10.9)	35 923 (13.6)	35 448 (9.0)	29 704 (14.0)	7193 (14.0)	3567 (10.9)	7681 (12.2)	2652 (13.3)	5878 (9.9)	1607 (11.9)	13 089 (6.4)
70-74	53 285 (8.1)	24 598 (9.3)	28 687 (7.3)	19 207 (9.1)	5848 (11.4)	2716 (8.3)	5925 (9.4)	2675 (13.4)	5819 (9.8)	1599 (11.8)	9496 (4.6)
75-79	40 390 (6.1)	16 241 (6.2)	24 149 (6.1)	11 610 (5.5)	4408 (8.6)	2055 (6.3)	4683 (7.5)	2576 (12.9)	6275 (10.5)	1557 (11.5)	7226 (3.5)
≥80	54 733 (8.3)	15 794 (6.0)	38 939 (9.9)	9201 (4.4)	4993 (9.7)	2608 (8.0)	6992 (11.1)	3985 (20.0)	14 069 (23.6)	3151 (23.3)	9734 (4.7)
Race and ethnicity^b^											
Asian/Pacific Islander	14 520 (2.2)	3009 (1.1)	11 511 (2.9)	2347 (1.1)	779 (1.5)	264 (0.8)	534 (0.9)	398 (2.0)	554 (0.9)	339 (2.5)	9305 (4.5)
Black	74 641 (11.4)	25 787 (9.8)	48 854 (12.4)	19 289 (9.1)	5307 (10.3)	4552 (13.9)	5188 (8.3)	1946 (9.8)	4915 (8.3)	1998 (14.8)	34 116 (15.3)
Hispanic	59 841 (9.1)	15 412 (5.8)	44 429 (11.3)	10 322 (4.9)	2304 (4.5)	2172 (6.6)	4223 (6.7)	2918 (14.6)	3067 (5.1)	1230 (9.1)	33 605 (16.3)
White	499 899 (76.1)	217 050 (82.2)	282 849 (71.9)	177 357 (83.9)	42 529 (82.7)	25 303 (77.4)	52 145 (83.1)	14 390 (72.2)	50 345 (84.5)	9806 (72.4)	128 024 (62.1)
Other/unknown	8416 (1.3)	2761 (1.0)	5655 (1.4)	2106 (1.0)	481 (0.9)	388 (1.2)	631 (1.0)	267 (1.3)	678 (1.1)	170 (1.3)	3695 (1.8)
Region											
Northeast	121 617 (18.5)	47 286 (17.9)	74 331 (18.9)	36 970 (17.5)	9806 (19.1)	6431 (19.7)	11 668 (18.6)	3885 (19.5)	12 657 (21.3)	2573 (19.0)	37 627 (18.3)
Midwest	143 240 (21.8)	57 356 (21.7)	85 884 (21.8)	46 588 (22.0)	11 492 (22.4)	6350 (19.4)	13 502 (21.5)	4418 (22.2)	15 185 (25.5)	2944 (21.7)	42 761 (20.8)
South	261 325 (39.8)	106 984 (40.5)	154 341 (39.2)	86 467 (40.9)	20 601 (40.1)	12 765 (39.1)	24 271 (38.7)	7752 (38.9)	21 786 (36.6)	5407 (39.9)	82 276 (39.9)
West	131 135 (20.0)	52 393 (19.8)	78 742 (20.0)	41 396 (19.6)	9501 (18.5)	7133 (21.8)	13 280 (21.2)	3864 (19.4)	9931 (16.7)	2619 (19.3)	43 411 (21.1)
Stage^c^											
Local	176 991 (26.9)	55 952 (21.2)	176 991 (45.0)	27 944 (13.2)	11 058 (21.5)	16 182 (49.5)	30 066 (47.9)	11 826 (59.4)	35 477 (59.6)	4894 (36.1)	95 496 (46.3)
Regional	147 297 (22.4)	156 487 (59.3)	147 297 (37.5)	140 233 (66.3)	30 046 (58.5)	9925 (30.4)	19 470 (31.0)	6329 (31.8)	18 895 (31.7)	5152 (38.0)	73 734 (35.8)
Distant	45 553 (6.9)	37 913 (14.4)	45 553 (11.6)	34 085 (16.1)	7508 (14.6)	3131 (9.6)	7007 (11.2)	697 (3.5)	2632 (4.4)	2056 (15.2)	26 350 (12.8)
Unknown	23 457 (3.6)	13 667 (5.2)	23 457 (6.0)	9159 (4.3)	2788 (5.4)	3441 (10.5)	6178 (9.8)	1067 (5.4)	2555 (4.3)	1441 (10.6)	10 495 (5.1)

Abbreviations: HPV, human papillomavirus; NA, not applicable; SCC, squamous cell carcinoma.

^a^ Percentages may not total 100 because of rounding.

^b^ Race and ethnicity self-reported; other/unknown included non-Hispanic American Indian/Alaska Native, other unspecified, or unknown.

^c^ Merged summary stage included local (localized only), regional (regional, direct extension only, regional lymph nodes only, direct extension and regional lymph nodes, or not otherwise specified), distant (distant site[s]/node[s]) involved), and unknown (not applicable, unknown, unstaged, unspecified, or death certificate-only).

### Overall Incidence of HPV-Associated Cancers

#### HPV-Associated Cancer in Women

In 2017, the overall incidence of HPV-associated cancers in women was 13.68 of 100 000. Of these cancers, 206 075 (52.4%) were attributed to cervical carcinoma, at 7.12 per 100 000. White women had the highest incidence of HPV-associated cancers, at 14.31 per 100 000; Asian/Pacific Islander women had the lowest incidence (7.57 per 100 000). Black women aged 80 years or older residing in the Northeast had the highest incidence of HPV-associated cancers, at 45.66 per 100 000.

#### HPV-Associated Cancer in Men

In 2017, the overall incidence of HPV-associated cancer in men was 11.00 per 100 000. Of these cancers, 211 421 (80.1%) were attributed to oropharyngeal cancer, at 8.89 per 100 000. White men had the highest incidence of HPV-associated cancers, at 12.50 per 100 000, compared with Asian/Pacific Islander men, who had the lowest incidence (2.92 per 100 000). The intersection of White men aged 60 to 64 years from the South had the highest incidence of HPV-associated cancers, 53.76 per 100 000.

### Trends in Incidence of Cervical Cancer

**Figure 1.**
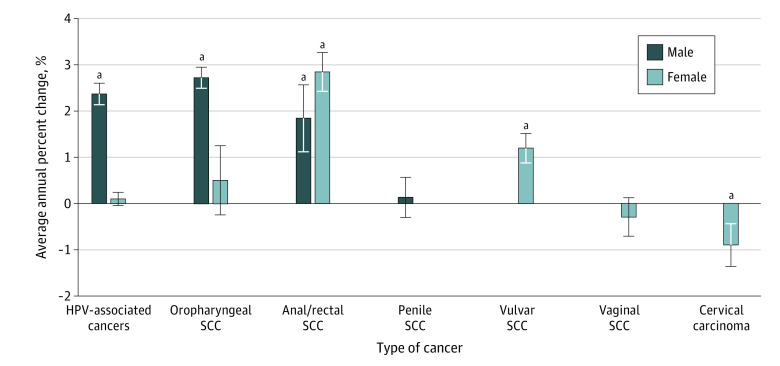
Average Annual Percent Change (AAPC) of Human Papillomavirus Virus (HPV)-Associated Cancers in US Cancer Statistics Public Use Databases From 2001 to 2017 Trends based on incidence were analyzed using the Joinpoint Regression Program, version 4.8.0.1, allowing up to 3 joinpoints. The AAPC is a summary measure of the trend over a prespecified fixed interval. It was computed as a weighted average of the APC from the joinpoint model, with the weights equal to the length of the APC interval. Vertical lines indicate the associated 95% CIs. SCC indicates squamous cell carcinoma. ^a^The AAPC is significantly different from 0 (*P* < .05).

**Figure 2.**
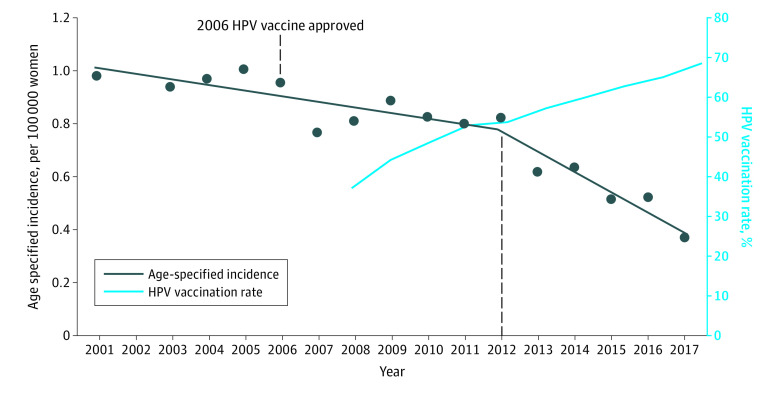
Age-Specified Incidences and Trends of Cervical Squamous Cell Carcinoma in Individuals Aged 20 to 24 Years and Human Papillomavirus Virus (HPV) Vaccination Rate (>1 Dose) in Adolescents Aged 13 to 17 Years Trends based on incidence were analyzed using the Joinpoint Regression Program, version 4.8.0.1, allowing up to 3 joinpoints. The average annual percent change (AAPC) is a summary measure of the trend over a prespecified fixed interval. It was computed as a weighted average of the APC from the joinpoint model, with the weights equal to the length of the APC interval. Vertical lines indicate the associated 95% CIs.

Over the past 17 years, the incidence of cervical carcinoma has decreased at an AAPC of 0.9% (*P* < .001) (Figure 1). Cervical carcinoma is decreasing in all race and ethnicity groups, with the largest decrease in Asian/Pacific Islander women, at an AAPC of −2.82% (*P* < .001). The incidence is decreasing in all regions in the US except the Midwest, at an AAPC of −0.62% (*P* < .001) (eTable 6 in the Supplement). We performed a subset analysis of cervical cancer incidence in girls aged 9 to 13 years in 2006, the year when the HPV vaccine was approved. These women were aged 20 to 24 years in 2017 at the end of our study. Before vaccine approval, cervical cancer rates were decreasing at 2.29% in the age 20- to 24-year group (*P* = .045). After the vaccine became available, the incidence of cervical cancer decreased significantly, at a rate of 9.50% annually in this same age group (*P* = .003). In the subset of SCC of the cervix, there was an 11.72% yearly decrease in new cases (*P* = .008) (Table 2; Figure 2). These trends were not observed in the older age groups. The demographic and clinical characteristics of the patients with cervical cancer are summarized in eTable 3 in the Supplement. Furthermore, after correcting for hysterectomy, the trends remained consistent (eTable 4 and eTable 5 in the Supplement).

**Table 2.** Comparison of Cervical Cancer Age-Specified Incidence and Trends in USCS

Age, y, per- cancer cell type	Incidence^a^					
	2001-2017, ASI	Trend 1		Trend 2		2001-2017, AAPC (95% CI)
		Years	APC (95% CI)	Year	APC (95% CI)	
20-24						
Cervical cancer	1.37 to 0.68	2001-2011	−2.29 (−4.45 to −0.07)^b^	2011-2017	−9.50 (−14.59 to −4.10)^b^	−5.05 (−7.24 to −2.82)^b^
Cervical carcinoma	1.34 to 0.60	2001-2017	−4.63 (−5.90 to −3.35)^b^	0	0	−4.63 (−5.90 to −3.35)^b^
Cervical SCC	0.98 to 0.37	2001-2012	−3.03 (−4.90 to −1.14)^b^	2012-2017	−11.72 (−18.89 to −3.92)^b^	−5.84 (−8.32 to −3.29)^b^
25-29						
Cervical cancer	6.79 to 4.47	2001-2017	−1.58 (−2.32 to −0.84)^b^	0	0	−1.58 (−2.32 to −0.84)^b^
Cervical carcinoma	6.71 to 4.33	2001-2017	−1.63 (−2.40 to −0.90)^b^	0	0	−1.63 (−2.40 to −0.90)^b^
Cervical SCC	4.72 to 3.17	2001-2005	−6.81 (−12.73 to −0.50)^b^	2005-2017	−0.28 (−1.52 to 0.97)	−1.96 (−3.61 to −0.28)^b^
30-34						
Cervical cancer	12.26 to 11.01	2001-2012	−2.15 (−2.69 to −1.62)^b^	2012-2017	2.53 (0.68 to 4.40)^b^	−0.72 (−1.32 to −0.11)^b^
Cervical carcinoma	12.14 to 10.84	2001-2012	−2.23 (−2.73 to −1.72)^b^	2012-2017	2.56 (0.82 to 4.34)^b^	−0.75 (−1.33 to −0.18)^b^
Cervical SCC	8.57 to 7.23	2001-2011	−3.31 (−4.00 to −2.62)^b^	2011-2017	2.76 (1.19 to 4.37)^b^	−1.08 (−1.73 to −0.43)^b^

Abbreviations: AAPC, average annual percent change; APC, annual percent change; ASI, age-specified incidence; SCC, squamous cell carcinoma; USCS, United States Cancer Statistics.

^a^ Trends based on incidence were analyzed using the Joinpoint Regression Program, version 4.8.0.1, allowing up to 3 joinpoints. The AAPC is a summary measure of the trend over a prespecified fixed interval. It was computed as a weighted average of the APC from the joinpoint model, with the weights equal to the length of the APC interval. Vertical lines indicate the associated 95% CIs.

^b^ The APC or AAPC is significantly different from 0 (*P* < .05).

### BRFSS System Data

#### Cervical Cancer Screening and HPV Vaccination

To evaluate whether the decrease in cervical cancer incidence is associated with changes in screening patterns, we used BRFSS data from 2001 to 2016. In this survey study, we found that cervical cancer screening rates in young women were declining or stable. In the group younger than 24 years, cervical cancer screening rates were declining at an AAPC of −3.61% (*P* < .001) (eTable 11 in the Supplement). In the same data set, we noted an increase in HPV vaccination rates in all age groups, with an AAPC of 23.4% (*P* < .001) (Table 3).

**Table 3.** HPV Vaccination Percentage With Trends in BRFSS and TeenVaxView

Variable	Incidence^a^					
	%^b^	Trend 1		Trend 2		AAPC (95% CI)^b^
		Years	APC (95% CI)	Year	APC (95% CI)	
BRFSS						
Age group (male plus female), y						
18-24	18.9 to 44.1	2008-2010	55.10 (2.83 to 133.95)^c^	2010-2016	2.24 (−4.62 to 9.60)	13.47 (4.61 to 23.08)^c^
25-29	4.8 to 33.2	2008-2010	96.77 (6.49 to 263.60)^c^	2010-2016	12.81 (1.68 to 25.14)^c^	29.64 (14.81 to 46.39)^c^
30-34	1.1 to 14.6	2008-2013	66.61 (45.33 to 91.02)^c^	2013-2016	7.77 (−20.62 to 46.29)	41.50 (27.91 to 56.52)^c^
TeenVaxView						
Age group 13-17 y (female)						
≥1 dose	37.2 to 69.9	2008-2010	15.23 (10.69 to 19.95)^c^	2010-2018	4.51 (4.06 to 4.97)^c^	6.57 (5.83 to 7.32)^c^
≥2 doses	28.3 to 57.4	2008-2010	19.91 (9.77 to 30.99)^c^	2010-2018	4.58 (3.58 to 5.60)^c^	7.48 (5.83 to 9.16)^c^
≥3 doses	17.9 to 37.9	2008-2010	35.00 (4.28 to 74.78)^c^	2010-2018	3.15 (0.29 to 6.10)^c^	8.85 (4.05 to 13.88)^c^

Abbreviations: AAPC, average annual percent change; APC, annual percent change; BRFSS, Behavioral Risk Factor Surveillance System; HPV, human papillomavirust.

^a^ Trends based on incidence were analyzed using the Joinpoint Regression Program, version 4.8.0.1, allowing up to 3 joinpoints. The AAPC is a summary measure of the trend over a prespecified fixed interval. It was computed as a weighted average of the APC from the joinpoint model, with the weights equal to the length of the APC interval. Vertical lines indicate the associated 95% CIs.

^b^ For BRFSS the percent years and AAPC years are 2008 to 2016, and for TeenVaxView, the percent years and AAPC years are 2008 to 2018.

^c^ The APC or AAPC is significantly different from zero (*P* < .05).

### TeenVaxView HPV

#### Vaccination of Adolescents

We then used the TeenVaxView data to study the vaccination rates of the adolescent population. Our data showed that the vaccination rate in adolescent females (aged 13-17 years) increased from 37.2% to 69.9% between 2008 and 2018, with an AAPC of 6.57% (95% CI, 5.83%-7.32%) (Table 3). A total of 66.3% of male adolescents had received at least 1 dose of the HPV vaccine. Vaccination rates were highest in Hispanic girls (75.3%) and lowest in White girls (66.1%).

### Trends in Incidence of Nonscreenable HPV-Associated Cancers

#### HPV-Associated Cancer in Women

During the past 17 years, a significant increase has occurred in the incidence of anal/rectal (AAPC, 2.83%) and vulvar (AAPC, 1.19%) cancer in women (*P* < .001 for all) (eTable 6 and eTable 7 in the Supplement). Anal/rectal cancers are increasing in both Black and White women. Regarding age, there is an increase in the incidence of anal/rectal cancer in all women older than 50 years, with the greatest increase in women aged 60 to 64 years (AAPC, 5.15%; *P* < .001). Vulvar cancer is increasing in all women older than 45 years, with the largest increase in women aged 60 to 64 years (AAPC, 3.06%; *P* < .001). Oropharyngeal and vaginal cancer rates remain stable.

#### HPV-Associated Cancer in Men

In men, there has also been a significant increase in HPV-associated cancers, with an AAPC of 2.36% per year (*P* < .001) (eTable 8 in the Supplement). Significant increases were noted in oropharyngeal (AAPC, 2.71%) and anal/rectal (1.83%) cancers (*P* < .001 for both). Oropharyngeal cancer is increasing in all regions of the US, with a significant increase in distant stage of disease (AAPC, 3.79%; *P* < .001). Oropharyngeal cancer was increasing in all men older than 50 years, with the largest increase in men aged 65 to 69 years (AAPC, 4.24%; *P* < .001). When examining new cases by race and ethnicity, we observed a decrease in Black men (AAPC, −1.35%) vs an increase in White men (AAPC, 3.48%) (*P* < .001) (eFigure, eTable 9, and eTable 10 in the Supplement). Anal/rectal cancer has increased in all racial and ethnic groups, with the largest increase in Black men (AAPC, 3.40%; *P* < .001). Penile cancer rates have remained stable.

## Discussion

In this study, we found differences in the incidence and trends of HPV-associated cancers. Most notably, cervical cancer rates have decreased. There are also sex disparities in HPV-associated cancers, with more than 80% of men with HPV-associated cancers diagnosed with oropharyngeal cancer—a nearly 5-fold higher incidence compared with women. In addition, the incidence of anal/rectal cancers is increasing in both men and women. The US Food and Drug Administration has approved the HPV vaccine owing to its efficacy in preventing cervical, oropharyngeal, and anal/rectal cancer. Although prior studies have evaluated the incidence of specific HPV-associated cancers, few studies have examined the trends of all HPV-associated cancers in association with screening and vaccination practices.^3^

It has been well established that the HPV vaccine reduces the incidence of cervical dysplasia based on randomized and prospective clinical trials.^18,19^ Our study found a decrease in invasive cervical cancer rates on a population level in the US, particularly in young women who were eligible for vaccination. The association of HPV vaccination with decreasing cervical cancer incidence, as well as the importance of early vaccination, has been reported in several countries, including Sweden, Finland, Denmark, and England, based on comprehensive national health registries combined with vaccination registries.^20,21,22,23,24^ In Sweden, Lei et al^20^ found that females younger than 17 years who were vaccinated had an incidence rate ratio of 0.12 for invasive cervical cancer compared with those never vaccinated. Using the US Cancer Statistics database, a population study found that, before vaccine approval, the incidence of cervical cancer was decreasing, at 1.7% annually; after the vaccine was approved in 2009, the rates decreased at 7.1% annually in women younger than 24 years. However, these authors did not evaluate the potential association of screening practices and vaccination rates in the US. In our study, we obtained survey data from the BRFSS and TeenVaxView and noted that cervical cancer screening rates are decreasing in young adults while HPV vaccination rates are increasing, particularly in adolescents. These findings suggest that HPV vaccination may have contributed to the decreasing incidence of cervical cancer in young adults. Because the US does not have a nationwide health care system in which residents have a singular identifier for all medical records, we are unable to directly attribute the decline in cervical cancer to vaccination. Nevertheless, even with a national electronic system, there are potential confounders, such as the healthy volunteer bias and other social determinants of health, including smoking, which can prevent more accurate estimation of the HPV vaccination and cancer trends.^25,26^ Furthermore, these data may be confounded by concurrent declines in sexual activity in young women, as reported in a study.^27^

Multiple studies have reported an increase in HPV-associated oropharyngeal cancer in various parts of the world.^28,29^ In the US, studies have noted that HPV-associated oropharyngeal cancer has been increasing over the past few decades, from 16% in 1984 to more than 70% in 2000.^30^ The HPV vaccine was approved in 2020 for the expanded indication to prevent oropharyngeal cancer. Because oropharyngeal cancers typically present 20 to 45 years after HPV-16 infection, with a median age at onset of 63 years, it may be too early to expect results from the vaccine.^31^ Modeling studies on the long-term outcome associated with HPV vaccination among men in the US suggest that, at the current rate of vaccination, the incidence of vaccine-type oropharyngeal cancers will remain high until the mid-2030s and then decrease and plateau after 2080. If 80% vaccination uptake is achieved (the Healthy People goal), a greater decrease in incidence would be expected after 2060.^32^ Unlike cervical cancer, an adequate or cost-effective screening test for oropharyngeal cancer does not exist.^30,33^ There are also sex differences in HPV prevalence and persistence. In this study, we found that more than 80% of new diagnoses of oropharyngeal cancer occurred in men, with increasing rates. An earlier study reported that men have a 2-fold higher risk of acquiring an oncogenic HPV oral infection compared with women and are less likely to clear oral HPV infections.^34^ The intersectional analyses of this report also identified certain at-risk populations that may benefit from screening. In addition, this analysis suggests the importance of vaccinating men as well as women against HPV, although additional research is needed to determine the efficacy of the HPV vaccine against oropharyngeal cancers.

Anal/rectal SCC is associated with sexual practices as well as HPV infection.^35,36,37^ Previous studies have noted an increase in anal/rectal SCC in the US with a greater increase in women compared with men.^38^ Our study also observed racial and ethnic disparities in the increasing incidence of anal/rectal cancer, with the highest increase in Black males. Previous studies on racial and ethnic disparities using National Inpatient Survey data also reported that Black males had a higher risk of developing cancer and Hispanic males had a lower risk.^39^ However, we noted an increasing incidence in all racial and ethnic groups, including Hispanic men. Anal/rectal cancer has a median age at onset of 62 years. When compared with cervical cancer, with a median age at onset of 49 years, this older age distribution may also account for the continued increase in this cancer and the failure to yet see the potential benefit of HPV vaccination. These findings on the increasing incidence of anal/rectal cancer highlight the importance of HPV vaccination as well as the need to develop more-effective screening strategies to prevent anal/rectal cancer, particularly in high-risk populations.

### Strengths and Limitations

This study has limitations. In addition to the retrospective design of the study, our results may be confounded by reporting bias, missing data, and lack of review of the central pathologic characteristics. This study is epidemiologic in nature, providing a large sample size to analyze modifiable risk factors on a population level. However, our analyses did not provide information on possible confounding factors, such as tobacco use and sexual behavior, that may affect our results. In addition, because a national registry of screening and vaccination does not exist in the US, we were only able to obtain information from national surveys to evaluate cancer screening and vaccination trends. Nevertheless, we believe that our cancer incidence is reliable because the USCS database represents 99% of the US population. To our knowledge, this is one of the only studies that used a cancer registry, cancer database, health behavioral survey, and vaccination registry to evaluate cancer incidence in association with screening practices and vaccination.

## Conclusions

Overall, the incidence of HPV-associated cancers is increasing. The results of this early report suggest that vaccination may be associated with a decrease in invasive cervical cancer on a population level. Our data also suggest that rates of HPV-associated oropharyngeal cancer may be increasing, particularly among men. This increase, along with that seen in anal/rectal cancer, may be attributed to a later median age of onset of this cancer and lower HPV vaccination rates in men. Although the HPV vaccine has been approved for more than 15 years in women, uptake has been slower than other recommended childhood vaccinations.^40^ Our results highlight the importance of the HPV vaccine. Continued efforts are needed to increase rates of vaccination as well as eliminate racial and ethnic disparities in cancer screening in the US.

## Notes

Supplement. **eTable 1.** Variable Definitions of HPV-Associated Cancers and Cervical Cancer in This Study **eTable 2.** Definitions of HPV-Associated Cancers and Cervical Cancer in USCS Public Use Databases From 2001 to 2017 **eTable 3.** Demographic and Clinical Characteristics of Cervical Cancer in USCS Public Use Databases From 2001 to 2017 **eTable 4.** Age-Specified Incidences and Trends of Female Cervical Cancer by Age Group and Cell Type in USCS Public Use Databases From 2001 to 2017 **eTable 5.** Age-Adjusted Incidences and Trends of HPV-Associated Cancers and Cervical Cancer in USCS Public Use Databases From 2001 to 2017 **eTable 6.** Age-Adjusted Incidences and Trends of Female HPV-Associated Cancers by Race and Ethnicity, Region, and Stage in USCS Public Use Databases From 2001 to 2017 **eTable 7.** Age-Specified Incidences and Trends of Female HPV-Associated Cancers by Age Group in USCS Public Use Databases From 2001 to 2017 **eTable 8.** Age-Adjusted Incidences and Trends of Male HPV-Associated Cancers by Race and Ethnicity, Region, and Stage in USCS Public Use Databases From 2001 to 2017 **eTable 9.** Age-Specified Incidences and Trends of Male HPV-Associated Cancers by Age Group in USCS Public Use Databases From 2001 to 2017 **eTable 10.** Age-Specified Incidences and Trends of Oropharyngeal SCC by Sex and Race and Ethnicity in USCS Public Use Databases From 2001 to 2017 **eTable 11.** Cervical Cancer Screening Trends in BRFSS (Ever Received any Cervical Cancer Screening) **eFigure.** Average Annual Percent Change in Oropharyngeal SCC by Sex in USCS Public Use Databases From 2001 to 2017
