# Supplementary material for: Trends in Human Papillomavirus–Associated Cancers, Demographic Characteristics, and Vaccinations in the US, 2001-2017
Source: JAMA Netw Open. 2022 Mar 16;5(3):e222530. doi: 10.1001/jamanetworkopen.2022.2530 (PMC8928005; doi:10.1001/jamanetworkopen.2022.2530)
Supplement: Supplement. — eTable 1. Variable Definitions of HPV-Associated Cancers and Cervical Cancer in This Study eTable 2. Definitions of HPV-Associated Cancers and Cervical Cancer in USCS Public Use Databases From 2001 to 2017 eTable 3. Demographic and Clinical Characteristics of Cervical Cancer in USCS Public Use Databases From 2001 to 2017 eTable 4. Age-Specified Incidences and Trends of Female Cervical Cancer by Age Group and Cell Type in USCS Public Use Databases From 2001 to 2017 eTable 5. Age-Adjusted Incidences and Trends of HPV-Associated Cancers and Cervical Cancer in USCS Public Use Databases From 2001 to 2017 eTable 6. Age-Adjusted Incidences and Trends of Female HPV-Associated Cancers by Race and Ethnicity, Region, and Stage in USCS Public Use Databases From 2001 to 2017 eTable 7. Age-Specified Incidences and Trends of Female HPV-Associated Cancers by Age Group in USCS Public Use Databases From 2001 to 2017 eTable 8. Age-Adjusted Incidences and Trends of Male HPV-Associated Cancers by Race and Ethnicity, Region, and Stage in USCS Public Use Databases From 2001 to 2017 eTable 9. Age-Specified Incidences and Trends of Male HPV-Associated Cancers by Age Group in USCS Public Use Databases From 2001 to 2017 eTable 10. Age-Specified Incidences and Trends of Oropharyngeal SCC by Sex and Race and Ethnicity in USCS Public Use Databases From 2001 to 2017 eTable 11. Cervical Cancer Screening Trends in BRFSS (Ever Received any Cervical Cancer Screening) eFigure. Average Annual Percent Change in Oropharyngeal SCC by Sex in USCS Public Use Databases From 2001 to 2017 [file jamanetwopen-e222530-s001.pdf]

Supplementary Online Content

Liao CI, Francoeur AA, Kapp DS, Caesar MAP, Huh WK, Chan JK. Trends in human papillomavirus–associated cancers, demographic characteristics, and vaccinations in the US, 2001-2017. *JAMA Netw Open*. 2022;5(3):e222530.
doi:10.1001/jamanetworkopen.2022.2530

eTable 1. Variable Definitions of HPV-Associated Cancers and Cervical Cancer in This Study

eTable 2. Definitions of HPV-Associated Cancers and Cervical Cancer in USCS Public Use Databases From 2001 to 2017

eTable 3. Demographic and Clinical Characteristics of Cervical Cancer in USCS Public Use Databases From 2001 to 2017

eTable 4. Age-Specified Incidences and Trends of Female Cervical Cancer by Age Group and Cell Type in USCS Public Use Databases From 2001 to 2017

eTable 5. Age-Adjusted Incidences and Trends of HPV-Associated Cancers and Cervical Cancer in USCS Public Use Databases From 2001 to 2017

eTable 6. Age-Adjusted Incidences and Trends of Female HPV-Associated Cancers by Race and Ethnicity, Region, and Stage in USCS Public Use Databases From 2001 to 2017

eTable 7. Age-Specified Incidences and Trends of Female HPV-Associated Cancers by Age Group in USCS Public Use Databases From 2001 to 2017

eTable 8. Age-Adjusted Incidences and Trends of Male HPV-Associated Cancers by Race and Ethnicity, Region, and Stage in USCS Public Use Databases From 2001 to 2017

eTable 9. Age-Specified Incidences and Trends of Male HPV-Associated Cancers by Age Group in USCS Public Use Databases From 2001 to 2017

eTable 10. Age-Specified Incidences and Trends of Oropharyngeal SCC by Sex and Race and Ethnicity in USCS Public Use Databases From 2001 to 2017

eTable 11. Cervical Cancer Screening Trends in BRFSS (Ever Received any Cervical Cancer Screening)

eFigure. Average Annual Percent Change in Oropharyngeal SCC by Sex in USCS Public Use Databases From 2001 to 2017

This supplementary material has been provided by the authors to give readers additional information about their work.

© 2022 Liao CI et al. *JAMA Network Open.*

eTable 1. Variable Definitions of HPV-Associated Cancers and Cervical Cancer in This Study

Database	Year	Sex	Race/Ethnic	Region	Stage [†]	Age Group
**USCS (Non-corrected)**	2001-2017	Male Female	NHW, NHB, Hispanic, NHAPI, Other/Unknown	Northeast South Midwest West	Local Regional Distant Unknown	0-19, 20-24, 25-29, 30-34, 35-39, 40-44, 45-49, 50-54, 55-59, 60-64, 65-69, 70-74, 75-79, 80+
**BRFSS**	2001-2016	Female	NHW, NHB, Hispanic, NHAPI, Other/Unknown	Northeast South Midwest West	-	18-24, 25-29, 30-34, 35-39, 40-44, 45-49, 50-54, 55-59, 60-64, 65-69, 70-74, 75-79, 80+
**TeenVaxView**	2008-2018	Male Female	NHW, NHB, Hispanic, NHAPI, NHAIAN	Northeast South Midwest West	-	13-17
**USCS (Corrected by hysterectomy and pregnancy)** [‡]	2001-2016	Female	NHW, NHB, Hispanic, NHAPI, Other/Unknown	-	-	0-19, 20-24, 25-29, 30-34, 35-39, 40-44, 45-49, 50-54, 55-59, 60-64, 65-69, 70-74, 75-79, 80+
**NCDB**	2001-2016	Female	NHW, NHB, Hispanic, NHAPI, Other/Unknown	-	-	0-19, 20-24, 25-29, 30-34, 35-39, 40-44, 45-49, 50-54, 55-59, 60-64, 65-69, 70-74, 75-79, 80+

© 2022 Liao CI et al. *JAMA Network Open.*

eTable 2. Definitions of HPV-Associated Cancers and Cervical Cancer in USCS Public Use Databases from 2001 to 2017†

Cancer Type	Sex	ICD-O-3 Site Codes	ICD-O-3 Histology Codes
**HPV-associated cancers**			
**Oropharyngeal SCC**	Male, Female	C01.9, C02.4, C02.8, C05.1, C05.2, C09.0, C09.1, C09.8, C09.9, C10.0, C10.1, C10.2, C10.3, C10.4, C10.8, C10.9, C14.0, C14.2, C14.8	8050-8084, 8120-8131
**Anal and rectal SCC**	Male, Female	C20.9, C21.0, C21.1, C21.2, C21.8	8050-8084, 8120-8131
**Vulvar SCC**	Female	C51.0, C51.1, C51.2, C51.8, C51.9	8050-8084, 8120-8131
**Vaginal SCC**	Female	C52.9	8050-8084, 8120-8131
**Penile SCC**	Male	C60.0, C60.1, C60.2, C60.8, C60.9	8050-8084, 8120-8131
**Cervical carcinoma**	Female	C53.0, C53.1, C53.8, C53.9	8010-8671, 8940-8941
**Cervical cancer**	Female	C53.0, C53.1, C53.8, C53.9	8000-9992 (excluded 9050-9055, 9140, 9590-9992)*
**Carcinoma**	Female	C53.0, C53.1, C53.8, C53.9	8010-8671, 8940-8941
**SCC**	Female	C53.0, C53.1, C53.8, C53.9	8050-8084, 8120-8131
**Adenocarcinoma**	Female	C53.0, C53.1, C53.8, C53.9	8140-8147, 8160-8162, 8180-8221, 8250-8507, 8514, 8520-8551, 8571-8574, 8576, 8940-8941
**Adeosquamous carcinoma**	Female	C53.0, C53.1, C53.8, C53.9	8560, 8570
**Other carcinoma**	Female	C53.0, C53.1, C53.8, C53.9	8010-8049, 8085-8119, 8132-8139, 8148-8159, 8163-8179, 8222-8249, 8508-8513, 8515-8519, 8552-8559, 8561-8569, 8575, 8577-8671

© 2022 Liao CI et al. *JAMA Network Open.*

**Non-carcinoma**	Female	C53.0, C53.1, C53.8, C53.9	8000-8009, 8672-8939, 8942-9992 (excluded 9050-9055, 9140, 9590-9992)*

Abbreviations: SCC, squamous cell carcinoma; ICD-O-3, International Classification of Diseases for Oncology, the third edition .
† All cases were microscopically confirmed and malignant behavior. (Reference 1)
*These cell types, including Kaposi sarcoma, mesothelioma, lymphomas, leukemias, myelomas, lymphoreticular, and immunoproliferative diseases (ICD-O-3 histology codes 9050-9055, 9140, 9590-9992), were excluded from this study. (Reference 2)
References 1. https://www.cdc.gov/cancer/uscs/public-use/predefined-seer-stat-variables.htm 2. https://seer.cancer.gov/data/

© 2022 Liao CI et al. *JAMA Network Open.*

eTable 3. Demographic and Clinical Characteristics of Cervical Cancer in USCS Public Use Databases from 2001 to 2017†

	Cervical Cancer	Carcinoma	SCC	Adenocarcinoma	Adenosquamous Carcinoma	Other Carcinoma	Non-Carcinoma
**Case Number (%)**	210776 (100)	206075 (97.8)	142390 (67.6)	47171 (22.4)	7013 (3.3)	9501 (4.5)	4701 (2.2)
**Age Group**	N (%)	N (%)	N (%)	N (%)	N (%)	N (%)	N (%)
**0-19**	295 (0.1)	185 (0.1)	66 (0.0)	81 (0.2)	-	29 (0.3)	110 (2.3)
**20-24**	2130 (1.0)	2011 (1.0)	1427 (1.0)	327 (0.7)	64 (0.9)	193 (2.0)	119 (2.5)
**25-29**	9384 (4.5)	9201 (4.5)	6430 (4.5)	1928 (4.1)	296 (4.2)	547 (5.8)	183 (3.9)
**30-34**	18757 (8.9)	18513 (9.0)	12472 (8.8)	4579 (9.7)	682 (9.7)	780 (8.2)	244 (5.2)
**35-39**	24469 (11.6)	24158 (11.7)	16024 (11.3)	6336 (13.4)	950 (13.5)	848 (8.9)	311 (6.6)
**40-44**	27852 (13.2)	27447 (13.3)	18581 (13.0)	6806 (14.4)	1077 (15.4)	983 (10.3)	405 (8.6)
**45-49**	26149 (12.4)	25627 (12.4)	17695 (12.4)	6034 (12.8)	947 (13.5)	951 (10.0)	522 (11.1)
**50-54**	23188 (11.0)	22720 (11.0)	16223 (11.4)	4785 (10.1)	785 (11.2)	927 (9.8)	468 (10.0)
**55-59**	20708 (9.8)	20235 (9.8)	14512 (10.2)	4131 (8.8)	669 (9.5)	923 (9.7)	473 (10.1)
**60-64**	16850 (8.0)	16433 (8.0)	11536 (8.1)	3567 (7.6)	501 (7.1)	829 (8.7)	417 (8.9)
**65-69**	13489 (6.4)	13089 (6.4)	9134 (6.4)	2830 (6.0)	380 (5.4)	745 (7.8)	400 (8.5)
**70-74**	9833 (4.7)	9496 (4.6)	6554 (4.6)	2120 (4.5)	257 (3.7)	565 (5.9)	337 (7.2)
**75-79**	7484 (3.6)	7226 (3.5)	5048 (3.5)	1548 (3.3)	180 (2.6)	450 (4.7)	258 (5.5)
**80+**	10188 (4.8)	9734 (4.7)	6688 (4.7)	2099 (4.4)	216 (3.1)	731 (7.7)	454 (9.7)
**Race/Ethnicity**	N (%)	N (%)	N (%)	N (%)	N (%)	N (%)	N (%)
**NHW**	130776 (62.0)	128024 (62.1)	84928 (59.6)	33017 (70.0)	4436 (63.3)	5643 (59.4)	2752 (58.5)

© 2022 Liao CI et al. *JAMA Network Open.*

**NHB**	32389 (15.4)	34116 (15.3)	24733 (17.4)	4208 (8.9)	871 (12.4)	1634 (17.2)	943 (20.1)
**Hispanic**	34329 (16.3)	33605 (16.3)	23913 (16.8)	6874 (14.6)	1226 (17.5)	1592 (16.8)	724 (15.4)
**NHAPI**	9456 (4.5)	9305 (4.5)	6241 (4.4)	2229 (4.7)	369 (5.3)	466 (4.9)	151 (3.2)
**Other/Unknown**	3826 (1.8)	3695 (1.8)	2575 (1.8)	843 (1.8)	111 (1.6)	166 (1.7)	131 (2.8)
**Region**	N (%)	N (%)	N (%)	N (%)	N (%)	N (%)	N (%)
**Northeast**	38538 (18.3)	37627 (18.3)	25581 (18.0)	8914 (18.9)	1375 (19.6)	1757 (18.5)	911 (19.4)
**Midwest**	43526 (20.7)	42761 (20.8)	29656 (20.8)	9997 (21.2)	1458 (20.8)	1650 (17.4)	765 (16.3)
**South**	84446 (40.1)	82276 (39.9)	58484 (41.1)	17183 (36.4)	2501 (35.7)	4108 (43.2)	2170 (46.2)
**West**	44266 (21.0)	43411 (21.1)	28669 (20.1)	11077 (23.5)	1679 (23.9)	1986 (20.9)	855 (18.2)
**Stage**	N (%)	N (%)	N (%)	N (%)	N (%)	N (%)	N (%)
**Local**	96876 (46.0)	95496 (46.3)	61576 (43.2)	27801 (58.9)	3227 (46.0)	2892 (30.4)	1380 (29.4)
**Regional**	74711 (35.4)	73734 (35.8)	56959 (40.0)	11719 (24.8)	2595 (37.0)	2461 (25.9)	977 (20.8)
**Distant**	27102 (12.9)	26350 (12.8)	17867 (12.5)	4992 (10.6)	939 (13.4)	2552 (26.9)	752 (16.0)
**Unknown**	12087 (5.7)	10495 (5.1)	5988 (4.2)	2659 (5.6)	252 (3.6)	1596 (16.8)	1592 (33.9)

Abbreviations: SCC, squamous cell carcinoma; NHW, Non-Hispanic White; NHB, Non-Hispanic Black; NHAPI, Non-Hispanic Asians or Pacific Islander; Other/Unknown (included Non-Hispanic American Indian/Alaska Native, other unspecified, or Unknown); NOS, not otherwise specified.
† Percentages may not total 100 because of rounding.
§ Merged summary stage included local (localized only), regional (regional, direct extension only, regional lymph nodes only, direct extension and regional lymph nodes, or NOS), distant (distant site(s)/node(s) involved), and unknown (not applicable, unknown, unstaged, unspecified, or death certificate-only).

© 2022 Liao CI et al. *JAMA Network Open.*

eTable 4. Age-Specified Incidences and Trends of Female Cervical Cancer by Age Group and Cell Type in USCS Public Use Databases from 2001 to 2017†

	ASI	Trend 1		Trend 2		Trend 3		Trend 4		2001-2017
**Age Group**	**2001 to 2017**	**Years**	**APC (95% CI)**	**Year**	**APC (95% CI)**	**Year**	**APC (95% CI)**	**Year**	**APC (95% CI)**	**AAPC (95% CI)**
**Cervical Cancer**										
**20-24**	1.37 to 0.68	2001-2011	-2.29* (-4.45 to -0.07)	2011-2017	-9.50* (-14.59 to -4.10)					-5.05* (-7.24 to -2.82)
**25-29**	6.79 to 4.47	2001-2017	-1.58* (-2.32 to -0.84)							-1.58* (-2.32 to -0.84)
**30-34**	12.26 to 11.01	2001-2012	-2.15* (-2.69 to -1.62)	2012-2017	2.53* (0.68 to 4.40)					-0.72* (-1.32 to -0.11)
**35-39**	14.34 to 13.49	2001-2017	-0.32 (-0.80 to -0.17)							-0.32 (-0.80 to -0.17)
**40-44**	16.43 to 14.84	2001-2017	-0.28 (-0.71 to 0.15)							-0.28 (-0.71 to 0.15)
**45-49**	15.09 to 13.02	2001-2017	-0.36 (-0.76 to 0.03)							-0.36 (-0.76 to 0.03)
**50-54**	14.38 to 12.44	2001-2006	-2.23* (-3.82 to -0.62)	2006-2017	0.01 (-0.48 to 0.49)					-0.70* (-1.24 to -0.16)
**55-59**	14.34 to 11.79	2001-2005	-3.85 (-7.24 to -0.34)	2005-2017	-0.29 (-0.91 to 0.32)					-1.20* (-2.09 to -0.30)
**60-64**	14.57 to 11.20	2001-2003	-6.40 (-14.4 to 2.29)	2003-2017	-0.98* (-1.36 to -0.61)					-1.68* (-2.69 to -0.65)
**65-69**	15.47 to 10.19	2001-2017	-2.38* (-2.85 to -1.92)							-2.38* (-2.85 to -1.92)
**70-74**	13.50 to 9.67	2001-2017	-2.14* (-2.56 to -1.72)							-2.14* (-2.56 to -1.72)
**75-80**	11.85 to 9.12	2001-2017	-1.96* (-2.45 to -1.47)							-1.96* (-2.45 to -1.47)
**80+**	11.10 to 7.27	2001-2013	-3.38* (-3.95 to -2.82)	2013-2017	0.03 (-3.28 to 3.46)					-2.54* (-3.37 to -1.71)
**Cervical Carcinoma**										
**20-24**	1.34 to 0.60	2001-2017	-4.63* (-5.90 to -3.35)							-4.63* (-5.90 to -3.35)

© 2022 Liao CI et al. *JAMA Network Open.*

**25-29**	6.71 to 4.33	2001-2017	-1.63* (-2.40 to -0.90)							-1.63* (-2.40 to -0.90)
**30-34**	12.14 to 10.84	2001-2012	-2.23* (-2.73 to -1.72)	2012-2017	2.56* (0.82 to 4.34)					-0.75* (-1.33 to -0.18)
**35-39**	14.23 to 13.22	2001-2017	-0.35 (-0.83 to 0.13)							-0.35 (-0.83 to 0.13)
**40-44**	16.29 to 14.56	2001-2017	-0.34 (-0.77 to 0.09)							-0.34 (-0.77 to 0.09)
**45-49**	14.83 to 12.74	2001-2017	-0.39 (-0.79 to 0.01)							-0.39 (-0.79 to 0.01)
**50-54**	14.16 to 12.23	2001-2003	-5.55 (-12.69 to 2.18)	2003-2017	-0.29 (-0.65 to 0.07)					-0.96* (-1.88 to -0.04)
**55-59**	14.13 to 11.53	2001-2005	-3.90* (-7.22 to -0.46)	2005-2017	-0.33 (-0.93 to 0.28)					-1.23* (-2.11 to -0.35)
**60-64**	14.36 to 10.88	2001-2003	-6.54 (-14.97 to 2.73)	2003-2017	-1.10* (-1.50 to -0.70)					-1.79* (-2.88 to -0.70)
**65-69**	15.08 to 9.93	2001-2017	-2.44* (-2.92 to -1.96)							-2.44* (-2.92 to -1.96)
**70-74**	13.09 to 9.19	2001-2017	-2.27* (-2.70 to -1.84)							-2.27* (-2.70 to -1.84)
**75-80**	11.58 to 8.85	2001-2017	-2.07* (-2.57 to -1.56)							-2.07* (-2.57 to -1.56)
**80+**	10.70 to 6.95	2001-2012	-3.58* (-4.22 to -2.94)	2012-2017	-0.63 (-2.98 to 1.78)					-2.67* (-3.43 to -1.90)
**Cervical SCC**										
**20-24**	0.98 to 0.37	2001-2012	-3.03* (-4.90 to -1.14)	2012-2017	-11.72* (-18.89 to -3.92)					-5.84* (-8.32 to -3.29)
**25-29**	4.72 to 3.17	2001-2005	-6.81* (-12.73 to -0.50)	2005-2017	-0.28 (-1.52 to 0.97)					-1.96* (-3.61 to -0.28)
**30-34**	8.57 to 7.23	2001-2011	-3.31* (-4.00 to -2.62)	2011-2017	2.76* (1.19 to 4.37)					-1.08* (-1.73 tp -0.43)
**35-39**	10.18 to 8.61	2001-2017	-1.15* (-1.68 to -0.62)							-1.15* (-1.68 to -0.62)
**40-44**	12.01 to 9.33	2001-2017	-1.44* (-1.93 to -0.94)							-1.44* (-1.93 to -0.94)
**45-49**	10.90 to 7.94	2001-2017	-1.21* (-1.69 to -0.73)							-1.21* (-1.69 to -0.73)

© 2022 Liao CI et al. *JAMA Network Open.*

**50-54**	10.43 to 8.39	2001-2017	-0.91* (-1.31 to -0.51)							-0.91* (-1.31 to -0.51)
**55-59**	10.49 to 8.29	2001-2006	-3.78* (-5.90 to -1.61)	2006-2017	-0.38 (-1.00 to 0.25)					-1.45* (-2.18 to -0.72)
**60-64**	10.91 to 7.32	2001-2003	-8.45* (-14.92 to -1.50)	2003-2006	0.08 (-7.04 to 7.76)	2006-2011	-3.81* (-5.90 to -1.68)	2011-2017	-0.27 (-1.39 to 0.87)	-2.37* (-3.81 to -0.92)
**65-69**	10.73 to 6.52	2001-2017	-3.18* (-3.73 to -2.62)							-3.18* (-3.73 to -2.62)
**70-74**	9.43 to 5.88	2001-2003	-8.09* (-13.72 to -2.08)	2003-2010	-0.72 (-1.86 to 0.43)	2010-2013	-6.17 (-12.44 to 0.56)	2013-2017	-1.90 (-3.98 to 0.22)	-3.00* (-4.30 to -1.68)
**75-80**	8.05 to 5.91	2001-2017	-2.31* (-2.89 to -1.72)							-2.31* (-2.89 to -1.72)
**80+**	7.23 to 5.02	2001-2013	-3.51* (-4.18 to -2.83)	2013-2017	1.22 (-2.78 to 5.38)					-2.35* (-3.44 to -1.34)
**Cervical Adenocarcinoma**										
**25-29**	1.27 to 0.80	2001-2017	-1.25 (-2.48 to 0.00)							-1.25 (-2.48 to 0.00)
**30-34**	2.52 to 2.88	2001-2009	2.55* (0.82 to 4.31)	2009-2012	-6.33 (-19.76 to 9.35)	2012-2017	3.59* (0.21 to 7.09)			1.14 (-1.62 to 3.98)
**35-39**	2.79 to 3.82	2001-2007	6.48* (3.04 to 10.04)	2007-2017	0.47 (-0.90 to 1.87)					2.69* (1.31 to 4.08)
**40-44**	3.13 to 4.33	2001-2017	3.14* (2.52 to 3.77)							3.14* (2.52 to 3.77)
**45-49**	2.46 to 3.82	2001-2017	2.67* (2.07 to 3.28)							2.67* (2.07 to 3.28)
**50-54**	2.51 to 2.91	2001-2017	1.18* (0.63 to 1.73)							1.18* (0.63 to 1.73)
**55-59**	2.47 to 2.51	2001-2017	0.63 (-0.08 to 1.35)							0.63 (-0.08 to 1.35)
**60-64**	2.34 to 2.51	2001-2017	1.27* (0.53 to 2.01)							1.27* (0.53 to 2.01)
**65-69**	2.68 to 2.49	2001-2017	0.43 (-0.48 to 1.34)							0.43 (-0.48 to 1.34)
**70-74**	2.74 to 2.45	2001-2017	-0.95* (-1.67 to -0.21)							-0.95* (-1.67 to -0.21)
**75-80**	2.56 to 2.17	2001-2017	-1.11 (-2.26 to 0.06)							-1.11 (-2.26 to 0.06)

© 2022 Liao CI et al. *JAMA Network Open.*

**80+**	2.12 to 1.34	2001-2017	-2.85* (-3.77 to -1.93)							-2.85* (-3.77 to -1.93)
**Cervical Adenosquamous carcinoma**										
**30-34**	0.53 to 0.30	2001-2017	-3.77* (-5.35 to -2.17)							-3.77* (-5.35 to -2.17)
**35-39**	0.66 to 0.42	2001-2007	5.28 (-0.67 to 11.59)	2007-2017	-6.33* (-9.01 to -3.57)					-2.13 (-4.60 to 0.40)
**40-44**	0.59 to 0.43	2001-2017	-1.87* (-3.32 to -0.39)							-1.87* (-3.32 to -0.39)
**45-49**	0.71 to 0.53	2001-2010	-5.26* (-7.60 to -2.86)	2010-2017	3.41 (-0.58 to 7.56)					-1.56 (-3.51 to 0.43)
**50-54**	0.66 to 0.35	2001-2017	-4.72* (-6.22 to -3.19)							-4.72* (-6.22 to -3.19)
**55-59**	0.50 to 0.32	2001-2017	-2.90* (-4.50 to -1.27)							-2.90* (-4.50 to -1.27)
**60-64**	0.47 to 0.23	2001-2017	-2.40* (-4.00 to -0.77)							-2.40* (-4.00 to -0.77)
**Cervical Other Carcinoma**										
**25-29**	0.40 to 0.30	2001-2017	-2.77* (-4.60 to -0.91)							-2.77* (-4.60 to -0.91)
**30-34**	0.52 to 0.42	2001-2017	-2.45* (-4.02 to -0.86)							-2.45* (-4.02 to -0.86)
**35-39**	0.60 to 0.36	2001-2017	-2.15* (-3.43 to -0.86)							-2.15* (-3.43 to -0.86)
**40-44**	0.55 to 0.48	2001-2017	-1.59* (-2.66 to -0.50)							-1.59* (-2.66 to -0.50)
**45-49**	0.76 to 0.45	2001-2017	-2.78* (-4.23 to -1.30)							-2.78* (-4.23 to -1.30)
**50-54**	0.56 to 0.57	2001-2017	-0.45 (-1.71 to 0.83)							-0.45 (-1.71 to 0.83)
**55-59**	0.67 to 0.42	2001-2017	-1.87* (-2.95 to -0.77)							-1.87* (-2.95 to -0.77)
**60-64**	0.64 to 0.51	2001-2017	-0.78 (-1.85 to 0.31)							-0.78 (-1.85 to 0.31)
**65-69**	1.14 to 0.68	2001-2006	-10.53* (-16.95 to -3.61)	2006-2017	-0.06 (-2.29 to 2.21)					-3.46* (-5.86 to -1.00)

© 2022 Liao CI et al. *JAMA Network Open.*

**70-74**	0.58 to 0.62	2001-2017	-2.08* (-3.94 to -0.18)							-2.08* (-3.94 to -0.18)
**75-80**	0.58 to 0.66	2001-2017	-1.17 (-3.31 to 1.03)							-1.17 (-3.31 to 1.03)
**80+**	0.92 to 0.45	2001-2017	-3.65* (-5.23 to -2.04)							-3.65* (-5.23 to -2.04)
**Cervical Non-Carcinoma**										
**40-44**	0.14 to 0.28	2001-2017	3.76* (1.59 to 5.98)							3.76* (1.59 to 5.98)
**45-49**	0.26 to 0.28	2001-2017	1.05 (-0.53 to 2.65)							1.05 (-0.53 to 2.65)
**80+**	0.31 to 0.32	2001-2017	-1.52 (-4.14 to 1.18)							-1.52 (-4.14 to 1.18)

Abbreviations: SCC, squamous cell carcinoma, ASI, age-specified incidence; APC, annual percent change; AAPC, average annual percent change

† Trends based on incidence was analyzed using the Joinpoint Regression Program, version 4.8.0.1, allowing up to 3 joinpoints. AAPC is a summary measure of the trend over a pre-specified fixed interval. It was computed as a weighted average of the APC from the joinpoint model, with the weights equal to the length the APC interval.

* The APC or AAPC is significantly different from zero ($p < .05$).

© 2022 Liao CI et al. *JAMA Network Open.*

eTable 5. Age-Adjusted Incidences and Trends of HPV-Associated Cancers and Cervical Cancer in USCS Public Use Databases from 2001 to 2017†

		AAI	Trend 1		Trend 2		2001-2017
**Cancer and Cell Type**	**N**	**2001 to 2017**	**Years**	**APC (95% CI)**	**Year**	**APC (95% CI)**	**AAPC (95% CI)**
**Male HPV associated cancers**	264019	7.71 to 11.00	2001-2017	2.36* (2.13 to 2.59)	-	-	2.36* (2.13 to 2.59)
**Oropharyngeal SCC**	211421	5.90 to 8.89	2001-2017	2.71* (2.48 to 2.93)	-	-	2.71* (2.48 to 2.93)
**Anal and rectal SCC**	32679	0.99 to 1.31	2001-2009	3.35* (2.13 to 4.59)	2009-2017	0.34 (-0.68 to 1.36)	1.83* (1.12 to 2.55)
**Penile SCC**	19919	0.82 to 0.80	2001-2017	0.13 (-0.30 to 0.56)	-	-	0.13 (-0.30 to 0.56)
**Female HPV associated cancers**	393298	13.96 to 13.68	2001-2017	0.09 (-0.05 to 0.24)			0.09 (-0.05 to 0.24)
**Oropharyngeal SCC**	51400	1.58 to 1.68	2001-2015	1.05* (0.72 to 1.37)	2015-2017	-3.30 (-9.13 to 29.2)	0.49 (-0.25 to 1.24)
**Anal and rectal SCC**	62721	1.53 to 2.37	2001-2009	3.71* (2.99 to 4.44)	2009-2017	1.96* (1.37 to 2.54)	2.83* (2.41 to 3.25)
**Vulvar SCC**	59559	1.72 to 2.09	2001-2012	1.73* (1.41 to 2.05)	2012-2017	0.01 (-0.92 to 0.94)	1.19* (0.86 to 1.52)
**Vaginal SCC**	13543	0.46 to 0.42	2001-2017	-0.30 (-0.72 to 0.12)			-0.30 (-0.72 to 0.12)
**Cervical carcinoma**	206075	8.68 to 7.12	2001-2012	-1.46* (-1.86 to -1.06)	2012-2017	0.34 (-1.05 to 1.74)	-0.90* (-1.36 to -0.44)
**Cervical Cancer (Uncorrected)**	210776	8.81 to 7.30	2001-2012	-1.40* (-1.79 to -1.00)	2012-2017	0.36 (-0.99 to 1.74)	-0.85* (-1.30 to -0.40)
**Carcinoma**	206075	8.68 to 7.12	2001-2012	-1.46* (-1.86 to -1.06)	2012-2017	0.34 (-1.05 to 1.74)	-0.90* (-1.36 to -0.44)
**SCC**	142390	6.28 to 4.73	2001-2012	-2.17* (-2.56 to -1.77)	2012-2017	0.19 (-1.21 to 1.61)	-1.44* (-1.90 to -0.98)
**Adenocarcinoma**	47171	1.62 to 1.89	2001-2017	1.28* (1.00 to 1.56)	-	-	1.28* (1.00 to 1.56)
**Adenosquamous carcinoma**	7013	0.35 to 0.21	2001-2017	-3.11* (-3.67 to -2.55)	-	-	-3.11* (-3.67 to -2.55)

© 2022 Liao CI et al. *JAMA Network Open.*

**Other carcinoma**	9501	0.42 to 0.31	2001-2017	-2.15* (-2.73 to -1.57)	-	-	-2.15* (-2.73 to -1.57)
**Non-Carcinoma**	4701	0.14 to 0.18	2001-2017	1.54* (0.96 to 2.12)	-	-	1.54* (0.96 to 2.12)
		**AAI**	**Trend 1**		**Trend 2**		**2001-2016**
**Cancer and Cell Type**	**N**	**2001 to 2016**	**Years**	**APC (95% CI)**	**Year**	**APC (95% CI)**	**AAPC (95% CI)**
**Cervical Cancer (Corrected)**	201609	12.66 to 10.04	2001-2016	-1.48* (-1.76 to -1.20)			-1.48* (-1.76 to -1.20)
**Carcinoma**	199239	12.54 to 9.90	2001-2016	-1.50* (-1.78 to -1.22)			-1.50* (-1.78 to -1.22)
**SCC**	134582	8.91 to 6.45	2001-2016	-2.12* (-2.42 to -1.81)			-2.12* (-2.42 to -1.81)
**Adenocarcinoma**	36415	2.00 to 1.99	2001-2016	0.07 (-0.24 to 0.38)			0.07 (-0.24 to 0.38)
**Adenosquamous carcinoma**	6599	0.47 to 0.24	2001-2016	-3.88* (-4.96 to -2.79)			-3.88* (-4.96 to -2.79)

Abbreviations: SCC, squamous cell carcinoma, AAI, age-adjusted incidence (adjusted to the 2000 US standard population); APC, annual percent change; AAPC.

† Trends based on incidence was analyzed using the Joinpoint Regression Program, version 4.8.0.1, allowing up to 3 joinpoints. AAPC is a summary measure of the trend over a pre-specified fixed interval. It was computed as a weighted average of the APC from the joinpoint model, with the weights equal to the length the APC interval.

‡ The age-adjusted and age-specified incidence in USCS were corrected by hysterectomy (age ≥ 18), pregnancy (age 20-45), and cervical cancer who received hysterectomy. (Reference 1)

* The APC or AAPC is significantly different from zero ($p < .05$).

Reference

1. Mary Kathryn Abel, Racial disparities in high-risk uterine cancer histologic subtypes: A United States Cancer Statistics study. Gynecol Oncol. 2021 May;161(2):470-476

© 2022 Liao CI et al. *JAMA Network Open.*

eTable 6. Age-Adjusted Incidences and Trends of Female HPV-Associated Cancers by Race and Ethnicity, Region, and Stage in USCS Public Use Databases from 2001 to 2017†

	AAI	Trend 1		Trend 2		Trend 3		Trend 4		2001-2017
	**2001 to 2017**	**Years**	**APC (95% CI)**	**Year**	**APC (95% CI)**	**Year**	**APC (95% CI)**	**Year**	**APC (95% CI)**	**AAPC (95% CI)**
**Female HPV associated cancers**	13.96 to 13.68	2001-2017	0.09 (-0.05 to 0.24)							0.09 (-0.05 to 0.24)
**Race/Ethnicity**										
**NHW**	13.19 to 14.31	2001-2017	0.78* (0.61 to 0.94)							0.78* (0.61 to 0.94)
**NHB**	17.26 to 13.27	2001-2017	-1.51* (-1.73 to -1.30)							-1.51* (-1.73 to -1.30)
**Hispanic**	18.37 to 12.49	2001-2010	-3.05* (-3.72 to -2.38)	2010-2017	-0.88 (-1.79 to 0.04)					-2.11* (-2.60 to -1.61)
**NHAPI**	11.06 to 7.57	2001-2003	-9.73* (-16.12 to -2.84)	2003-2006	0.53 (-6.47 to 8.06)	2006-2009	-4.59 (-10.98 to 2.26)	2009-2017	-0.44 (-1.13 to 0.26)	-2.25* (-3.90 to -0.58)
**Other or Unknown**	25.71 to 36.93	2001-2017	1.32* (0.44 to 2.20)							1.32* (0.44 to 2.20)
**Region**										
**Northeast**	13.57 to 13.54	2001-2017	0.07 (-0.07 to 0.22)							0.07 (-0.07 to 0.22)
**Midwest**	13.32 to 13.96	2001-2017	0.54* (0.32 to 0.76)							0.54* (0.32 to 0.76)
**South**	15.11 to 14.49	2001-2017	0.08 (-0.13 to 0.28)							0.08 (-0.13 0.28)
**West**	13.12 to 12.14	2001-2017	-0.38* (-0.56 to -0.20)							-0.38* (-0.56 -0.20)
**Stage**										
**Local**	6.98 to 6.30	2001-2005	-3.35* (-5.34 to -1.31)	2005-2017	0.22 (-0.17 to 0.60)					-0.69* (-1.22 -0.15)
**Regional**	4.75 to 5.08	2001-2006	2.05* (0.41 to 3.72)	2006-2017	-0.08 (-0.53 to 0.37)					0.58* (0.04 1.12)
**Distant**	1.13 to 1.51	2001-2005	7.17* (3.12 to 11.38)	2005-2015	2.57* (1.62 to 3.52)	2015-2017	-10.59* (-19.13 to -1.15)			1.94* (0.46 to 3.43)

© 2022 Liao CI et al. *JAMA Network Open.*

**Unknown**	1.11 to 0.78	2001-2006	-7.23* (-12.08 to -2.11)	2006-2017	-0.44 (-2.14 to 1.28)					-2.62* (-4.40 to -0.80)
**Female Oropharyngeal SCC**	1.58 to 1.68	2001-2015	1.05* (0.72 to 1.37)	2015-2017	-3.30 (-9.13 to 29.2)					0.49 (-0.25 to 1.24)
**Race/Ethnicity**										
**NHW**	1.64 to 1.96	2001-2015	1.72* (1.40 to 2.05)	2015-2017	-2.71 (-8.64 to 3.60)					1.16* (0.40 to 1.92)
**NHB**	1.88 to 1.26	2001-2017	-1.86* (-2.31 to -1.40)							-1.86* (-2.31 to -1.40)
**Hispanic**	0.89 to 0.89	2001-2017	0.61 (-0.32 to 1.54)							0.61 (-0.32 to 1.54)
**NHAPI**	0.56 to 0.50	2001-2017	1.07 (-0.63 to 2.79)							1.07 (-0.63 to 2.79)
**Region**										
**Northeast**	1.58 to 1.78	2001-2017	1.04* (0.54 to 1.54)							1.04* (0.54 to 1.54)
**Midwest**	1.45 to 1.71	2001-2017	1.19* (0.61 to 1.77)							1.19* (0.61 to 1.77)
**South**	1.71 to 1.77	2001-2017	0.83* (0.44 to 1.23)							0.83* (0.44 to 1.23)
**West**	1.48 to 1.39	2001-2017	-0.14 (-0.45 to 0.16)							-0.14 (-0.45 to 0.16)
**Stage**										
**Local**	0.39 to 0.32	2001-2017	-0.89* (-1.39 to -0.39)							-0.89* (-1.39 to -0.39)
**Regional**	0.92 to 1.11	2001-2004	-2.82 (-6.49 to 1.00)	2004-2017	1.92* (1.55 to 2.29)					1.02* (0.31 to 1.73)
**Distant**	0.14 to 0.15	2001-2005	23.98* (10.42 to 39.20)	2005-2015	0.37 (-2.11 to 2.91)	2015-2017	-29.98* (-49.94 to -2.06)			1.15 (-3.41 to 5.94)
**Unknown**	0.13 to 0.10	2001-2013	-3.76* (-5.73 to -1.76)	2013-2017	9.97 (-1.03 to 22.19)					-0.50 (-3.20 to 2.27)
**Female Anal and Rectal SCC**	1.53 to 2.37	2001-2009	3.71* (2.99 to 4.44)	2009-2017	1.96* (1.37 to 2.54)					2.83* (2.41 to 3.25)
**Race/Ethnicity**										
**NHW**	1.63 to 2.77	2001-2008	4.52* (3.42 to 5.63)	2008-2017	2.68* (2.07 to 3.29)					3.48* (2.95 to 4.01)

© 2022 Liao CI et al. *JAMA Network Open.*

**NHB**	1.28 to 1.73	2001-2017	1.88* (1.38 to 2.38)							1.88* (1.38 to 2.38)
**Hispanic**	1.49 to 1.56	2001-2017	0.42 (-0.17 to 1.01)							0.42 (-0.17 to 1.01)
**NHAPI**	0.40 to 0.38	2001-2017	-1.30 (-3.80 to 1.28)							-1.30 (-3.80 to 1.28)
**Region**										
**Northeast**	1.49 to 2.38	2001-2017	3.06* (2.69 to 3.44)							3.06* (2.68 to 3.44)
**Midwest**	1.32 to 2.46	2001-2017	3.43* (2.93 to 3.94)							3.43* (2.93 to 3.94)
**South**	1.65 to 2.48	2001-2017	2.58* (2.15 to 3.01)							2.58* (2.15 to 3.01)
**West**	1.57 to 2.09	2001-2010	3.09* (2.11 to 4.08)	2010-2017	0.75 (-0.41 to 1.92)					2.06* (1.38 to 2.74)
**Stage**										
**Local**	0.80 to 1.03	2001-2017	1.79* (1.42 to 2.17)							1.79* (1.42 to 2.17)
**Regional**	0.39 to 0.90	2001-2009	7.02* (6.19 to 7.86)	2009-2012	-1.09 (-6.77 to 4.93)	2012-2017	6.93* (5.71 to 8.16)			5.42* (4.31 to 6.55)
**Distant**	0.12 to 0.24	2001-2004	17.94* (4.63 to 32.95)	2004-2015	4.86* (3.50 to 6.23)	2015-2017	-12.98 (-25.37 to 1.46)			4.72* (1.96 to 7.56)
**Unknown**	0.22 to 0.20	2001-2017	-0.54 (-1.08 to 0.01)							-0.54 (-1.08 to 0.01)
**Female Vulvar SCC**	1.72 to 2.09	2001-2012	1.73* (1.41 to 2.05)	2012-2017	0.01 (-0.92 to 0.94)					1.19* (0.86 to 1.52)
**Race/Ethnicity**										
**NHW**	1.87 to 2.40	2001-2014	2.12* (1.91 to 2.33)	2014-2017	-0.81 (-2.56 to 0.97)					1.56* (1.22 to 1.90)
**NHB**	1.37 to 1.75	2001-2017	1.07* (0.35 to 1.80)							1.07* (0.35 to 1.80)
**Hispanic**	1.27 to 1.16	2001-2017	-0.49 (-1.09 to 0.11)							-0.49 (-1.09 to 0.11)
**NHAPI**	0.39 to 0.48	2001-2017	1.14 (-0.52 to 2.83)							1.14 (-0.52 to 2.83)
**Other or Unknown**	3.06 to 4.46	2001-2017	0.88 (-1.02 to 2.81)							0.88 (-1.02 to 2.81)
**Region**										

© 2022 Liao CI et al. *JAMA Network Open.*

**Northeast**	1.84 to 2.26	2001-2017	1.36* (0.91 to 1.82)							1.36* (0.91 to 1.82)
**Midwest**	1.85 to 2.40	2001-2012	2.66* (2.08 to 3.25)	2012-2017	0.36 (-1.33 to 2.08)					1.94* (1.34 to 2.55)
**South**	1.74 to 2.09	2001-2017	1.22* (0.86 t0 1.58)							1.22* (0.86 to 1.58)
**West**	1.41 to 1.62	2001-2017	0.45 (-0.13 to 1.04)							0.45 (-0.13 to 1.04)
**Stage**										
**Local**	1.09 to 1.35	2001-2005	-1.86 (-5.17 to 1.57)	2005-2017	2.34* (1.76 to 2.93)					1.27* (0.40 to 2.15)
**Regional**	0.49 to 0.54	2001-2006	9.43* (3.63 to 15.55)	2006-2017	-2.25* (-3.67 to -0.80)					1.26 (-0.52 to 3.08)
**Distant**	0.05 to 0.10	2001-2014	7.32* (5.26 to 9.43)	2014-2017	-9.20 (-21.51 to 5.04)					4.01* (1.10 to 7.00)
**Unknown**	0.10 to 0.09	2001-2017	-0.68 (-2.46 to 1.13)							-0.68 (-2.46 to 1.13)
**Female Vaginal SCC**	0.46 to 0.42	2001-2017	-0.30 (-0.72 to 0.12)							-0.30 (-0.72 to 0.12)
**Race/Ethnicity**										
**NHW**	0.43 to 0.40	2001-2017	0.01 (-0.62 to 0.64)							0.01 (-0.62 to 0.64)
**NHB**	0.73 to 0.62	2001-2017	-1.14* (-1.74 to -0.53)							-1.14* (-1.74 to -0.53)
**Hispanic**	0.60 to 0.44	2001-2017	-1.72* (-2.78 to 0.64)							-1.72* (-2.78 to -0.64)
**Region**										
**Northeast**	0.44 to 0.38	2001-2017	-0.50 (-1.50 to 0.52)							-0.50 (-1.50 to 0.52)
**Midwest**	0.41 to 0.38	2001-2017	0.07 (-1.02 to 1.17)							0.07 (-1.02 to 1.17)
**South**	0.53 to 0.48	2001-2017	-0.39 (-1.04 to 0.27)							-0.39 (-1.04 to 0.27)
**West**	0.40 to 0.40	2001-2017	-0.39 (-1.19 to 0.42)							-0.39 (-1.19 to 0.42)
**Stage**										

© 2022 Liao CI et al. *JAMA Network Open.*

**Local**	0.20 to 0.17	2001-2005	-8.03* (-14.42 to -1.16)	2005-2017	-0.07 (-1.43 to 1.31)					-2.12* (-3.93 to -0.28)
**Regional**	0.14 to 0.15	2001-2017	0.49 (-0.49 to 1.48)							0.49 (-0.49 to 1.48)
**Distant**	0.04 to 0.06	2001-2012	3.93* (1.31 to 6.61)	2012-2017	-5.19 (-12.06 to 2.22)					0.99 (-1.64 to 3.69)
**Unknown**	0.07 to 0.05	2001-2004	-16.04* (-24.19 to -7.01)	2004-2010	4.18 (-0.85 to 9.46)	2010-2013	-8.72 (-26.73 to 13.72)	2013-2017	5.31 (-1.55 to 12.65)	-2.14 (-6.12 to 2.02)
**Female Cervical Carcinoma**	8.68 to 7.12	2001-2012	-1.46* (-1.86 to -1.06)	2012-2017	0.34 (-1.05 to 1.74)					-0.90* (-1.36 to 0.44)
**Race/Ethnicity**										
**NHW**	7.63 to 6.79	2001-2017	-0.49* (-0.78 to -0.20)							-0.49* (-0.78 to -0.20)
**NHB**	12.01 to 7.90	2001-2017	-2.41* (-2.72 to -2.11)							-2.41* (-2.72 to -2.11)
**Hispanic**	14.13 to 8.44	2001-2011	-4.04* (-4.77 to -3.30)	2011-2017	-0.73 (-2.27 to 0.83)					-2.81* (-3.47 to -2.15)
**NHAPI**	9.36 to 5.91	2001-2003	-11.88* (-19.27 to -3.81)	2003-2006	1.39 (-7.04 to 10.59)	2006-2009	-6.10 (-13.73 to 2.20)	2009-2017	-0.71 (-1.56 to 0.16)	-2.82* (-4.79 to -0.80)
**Other or Unknown**	17.64 to 23.60	2001-2017	0.69 (-0.27 to 1.66)							0.69 (-0.27 to 1.66)
**Region**										
**Northeast**	8.21 to 6.74	2001-2017	-1.26* (-1.50 to -1.02)							-1.26* (-1.50 to -1.02)
**Midwest**	8.28 to 7.01	2001-2012	-1.43* (-2.00 to -0.85)	2012-2017	1.19 (-0.86 to 3.27)					-0.62 (-1.29 to 0.06)
**South**	9.47 to 7.65	2001-2004	-3.56* (-6.64 to -0.38)	2004-2017	-0.65* (-0.99 to -0.31)					-1.20* (-1.80 to -0.61)
**West**	8.24 to 6.63	2001-2013	-1.66* (-2.15 to -1.16)	2013-2017	0.74 (-1.95 to 3.49)					-1.06* (-1.75 to -0.37)
**Stage**										
**Local**	4.50 to 3.43	2001-2004	-4.98* (-8.16 to -1.70)	2004-2013	-1.78* (-2.57 to -0.98)	2013-2017	1.99 (-0.40 to 4.44)			-1.47* (-2.30 to -0.62)
**Regional**	2.81 to 2.38	2001-2017	-0.92* (-1.29 -0.55)							-0.92* (-1.29 to -0.55)

© 2022 Liao CI et al. *JAMA Network Open.*

**Distant**	0.78 to 0.96	2001-2015	2.37* (2.04 to 2.69)	2015-2017	-4.82 (-10.52 to 1.26)					1.44* (0.69 to 2.19)
**Unknown**	0.60 to 0.34	2001-2006	-9.98* (-14.74 to -4.95)	2006-2017	-1.38 (-3.25 to 0.53)					-4.15* (-5.99 to -2.28)

Abbreviations: SCC, squamous cell carcinoma, AAI, age-adjusted incidence (adjusted to the 2000 US standard population); APC, annual percent change; AAPC, average annual percent change; NHW, Non-Hispanic White; NHB, Non-Hispanic Black; NHAPI, Non-Hispanic Asians or Pacific Islander; Other/Unknown (included Non-Hispanic American Indian/Alaska Native, Other unspecified, or Unknown).

† Trends based on incidence was analyzed using the Joinpoint Regression Program, version 4.8.0.1, allowing up to 3 joinpoints. AAPC is a summary measure of the trend over a pre-specified fixed interval. It was computed as a weighted average of the APC from the joinpoint model, with the weights equal to the length the APC interval.

§ Merged summary stage included local (localized only), regional (regional, direct extension only, regional lymph nodes only, direct extension and regional lymph nodes, or NOS), distant (distant site(s)/node(s) involved), and unknown (not applicable, unknown, unstaged, unspecified, or death certificate-only).

* The APC or AAPC is significantly different from zero ($p < .05$).

© 2022 Liao CI et al. *JAMA Network Open.*

eTable 7. Age-Specified Incidences and Trends of Female HPV-Associated Cancers by Age Group in USCS Public Use Databases from 2001 to 2017[†]

	ASI	Trend 1		Trend 2		Trend 3		Trend 4		2001-2017
**Age Group**	**2001 to 2017**	**Years**	**APC (95% CI)**	**Year**	**APC (95% CI)**	**Year**	**APC (95% CI)**	**Year**	**APC (95% CI)**	**AAPC (95% CI)**
**Female HPV associated cancer**										
**20-24**	1.40 to 0.62	2001-2012	-2.46* (-4.15 to -0.74)	2012-2017	-11.20* (-17.53 -4.38)					-5.28* (-7.48 to -3.03)
**25-29**	6.99 to 4.59	2001-2017	-1.58* (-2.29 to -0.86)							-1.58* (-2.29 to -0.86)
**30-34**	13.10 to 11.67	2001-2011	-2.33* (-2.91 to -1.75)	2011-2017	1.72* (0.41 to 3.05)					-0.83* (-1.38 to -0.28)
**35-39**	16.34 to 15.20	2001-2017	-0.40 (-0.82 to 0.02)							-0.40 (-0.82 to 0.02)
**40-44**	20.80 to 18.18	2001-2017	-0.57* (-0.88 to -0.25)							-0.57* (-0.88 to -0.25)
**45-49**	21.59 to 20.31	2001-2017	-0.10 (-0.45 to 0.26)							-0.10 (-0.45 to 0.26)
**50-54**	23.53 to 24.75	2001-2012	1.19* (0.71 to 1.66)	2012-2017	-0.71 (-2.16 to 0.76)					0.59* (0.09 to 1.10)
**55-59**	24.99 to 28.73	2001-2017	1.37* (1.06 to 1.68)							1.37* (1.06 to 1.68)
**60-64**	27.30 to 32.37	2001-2004	-0.85 (-2.83 to 1.17)	2004-2007	2.14 (-1.59 to 6.01)	2007-2010	0.00 (-3.32 to 3.44)	2010-2017	1.84* (1.43 to 2.24)	1.04* (0.21 to 1.88)
**65-69**	30.96 to 32.57	2001-2017	0.50* (0.18 to 0.82)							0.50* (0.18 to 0.82)
**70-74**	32.42 to 32.79	2001-2017	0.23 (-0.04 to 0.51)							0.23 (-0.04 to 0.51)
**75-80**	34.01 to 33.38	2001-2017	0.09 (-0.25 to 0.44)							0.09 (-0.25 to 0.44)
**80+**	34.21 to 31.93	2001-2017	-0.22* (-0.43 to -0.01)							-0.22* (-0.43 to -0.01)
**Female Oropharyngeal SCC**										
**35-39**	0.34 to 0.44	2001-2017	1.03 (-1.14 to 3.25)							1.03 (-1.14 to 3.25)

© 2022 Liao CI et al. *JAMA Network Open.*

**40-44**	1.03 to 0.60	2001-2017	-1.63* (-3.05 to -0.19)							-1.63* (-3.05 to -0.19)
**45-49**	1.75 to 1.78	2001-2004	8.49 (-3.33 to 21.74)	2004-2017	-0.92 (-2.05 to 0.22)					0.78 (-1.33 to 2.94)
**50-54**	3.02 to 3.38	2001-2013	3.17* (1.95 to 4.41)	2013-2017	-4.95 (-10.37 to 0.80)					1.08 (-0.47 to 2.66)
**55-59**	4.20 to 5.39	2001-2017	2.35* (1.76 to 2.94)							2.35* (1.76 to 2.94)
**60-64**	5.02 to 6.38	2001-2017	0.92* (0.35 to 1.49)							0.92* (0.35 to 1.49)
**65-69**	5.57 to 6.36	2001-2017	0.54 (-0.15 to 1.23)							0.54 (-0.15 to 1.23)
**70-74**	7.04 to 6.30	2001-2017	0.10 (-0.47 to 0.67)							0.10 (-0.47 to 0.67)
**75-80**	6.46 to 6.06	2001-2017	0.40 (-0.36 to 1.17)							0.40 (-0.36 to 1.17)
**80+**	4.31 to 4.30	2001-2017	0.94* (0.22 to 1.66)							0.94* (0.22 to 1.66)
**Female Anal and Rectal SCC**										
**35-39**	0.63 to 0.64	2001-2017	-0.73 (-2.49 to 1.06)							-0.73 (-2.49 to 1.06)
**40-44**	1.55 to 1.32	2001-2017	-1.33* (-2.06 to -0.59)							-1.33* (-2.06 to -0.59)
**45-49**	2.49 to 2.83	2001-2007	5.40* (2.75 to 8.12)	2007-2017	-2.03* (-3.12 to -0.92)					0.69 (-0.38 to 1.77)
**50-54**	3.23 to 4.73	2001-2009	6.41* (4.27 to 8.60)	2009-2017	-0.66 (-2.35 to 1.06)					2.82* (1.59 to 4.05)
**55-59**	3.11 to 7.34	2001-2009	7.17* (4.85 to 9.55)	2009-2017	2.87* (1.29 to 4.47)					5.00* (3.74 to 6.27)
**60-64**	4.16 to 8.95	2001-2017	5.15* (4.66 to 5.64)							5.15* (4.66 to 5.64)
**65-69**	4.37 to 8.97	2001-2017	5.02* (4.48 to 5.57)							5.02* (4.48 to 5.57)
**70-74**	4.61 to 8.42	2001-2017	3.27* (2.66 to 3.89)							3.27* (2.66 to 3.89)
**75-80**	5.33 to 7.57	2001-2017	2.47* (1.86 to 3.08)							2.47* (1.86 to 3.08)

© 2022 Liao CI et al. *JAMA Network Open.*

**80+**	5.53 to 6.36	2001-2017	1.23* (0.69 to 1.77)							1.23* (0.69 to 1.77)
**Female Vulvar SCC**										
**30-34**	0.40 to 0.43	2001-2017	0.05 (-1.54 to 1.67)							0.05 (-1.54 to 1.67)
**35-39**	0.96 to 0.81	2001-2017	-0.81* (-1.59 to -0.02)							-0.81* (-1.59 to -0.02)
**40-44**	1.72 to 1.36	2001-2017	-1.28* (-2.06 to -0.50)							-1.28* (-2.06 to -0.50)
**45-49**	2.06 to 2.56	2001-2007	4.46* (1.74 to 7.24)	2007-2017	-0.67 (-1.79 to 0.48)					1.23* (0.12 to 2.34)
**50-54**	2.44 to 3.82	2001-2017	2.46* (1.70 to 3.22)							2.46* (1.70 to 3.22)
**55-59**	2.70 to 3.62	2001-2017	3.00* (2.25 to 3.75							3.00* (2.25 to 3.75
**60-64**	2.84 to 5.07	2001-2017	3.06* (2.22 to 3.90)							3.06* (2.22 to 3.90)
**65-69**	4.37 to 5.88	2001-2017	1.88* (1.43 to 2.33)							1.88* (1.43 to 2.33)
**70-74**	5.80 to 7.33	2001-2017	1.51* (0.97 to 2.06)							1.51* (0.97 to 2.06)
**75-80**	8.49 to 8.58	2001-2017	0.73* (0.10 to 1.37)							0.73* (0.10 to 1.37)
**80+**	10.82 to 12.19	2001-2017	0.76* (0.41 to 1.11)							0.76* (0.41 to 1.11)
**Female Vaginal SCC**										
**40-44**	0.21 to 0.34	2001-2017	-0.66 (-2.55 to 1.26)							-0.66 (-2.55 to 1.26)
**45-49**	0.46 to 0.40	2001-2017	0.94 (-0.55 to 2.45)							0.94 (-0.55 to 2.45)
**50-54**	0.68 to 0.59	2001-2017	-0.94 (-2.01 to 0.14)							-0.94 (-2.01 to 0.14)
**55-59**	0.84 to 0.86	2001-2017	0.60 (-0.41 to 1.63)							0.60 (-0.41 to 1.63)
**60-64**	0.92 to 1.10	2001-2017	0.99 (-0.45 to 2.45)							0.99 (-0.45 to 2.45)

© 2022 Liao CI et al. *JAMA Network Open.*

**65-69**	1.57 to 1.43	2001-2017	-0.37 (-1.57 to 0.84)							-0.37 (-1.57 to 0.84)
**70-74**	1.87 to 1.55	2001-2017	0.30 (-0.79 to 1.40)							0.30 (-0.79 to 1.40)
**75-80**	2.17 to 2.32	2001-2017	-0.26 (-1.21 to 0.70)							-0.26 (-1.21 to 0.70)
**80+**	2.86 to 2.14	2001-2017	-1.11* (-1.66 to -0.57)							-1.11* (-1.66 to -0.57)
**Female Cervical Carcinoma**										
**20-24**	1.34 to 0.60	2001-2017	-4.63* (-5.90 to -3.35)							-4.63* (-5.90 to -3.35)
**25-29**	6.71 to 4.33	2001-2017	-1.63* (-2.40 to -0.90)							-1.63* (-2.40 to -0.90)
**30-34**	12.14 to 10.84	2001-2012	-2.23* (-2.73 to -1.72)	2012-2017	2.56* (0.82 to 4.34)					-0.75* (-1.33 to -0.18)
**35-39**	14.23 to 13.22	2001-2017	-0.35 (-0.83 to 0.13)							-0.35 (-0.83 to 0.13)
**40-44**	16.29 to 14.56	2001-2017	-0.34 (-0.77 to 0.09)							-0.34 (-0.77 to 0.09)
**45-49**	14.83 to 12.74	2001-2017	-0.39 (-0.79 to 0.01)							-0.39 (-0.79 to 0.01)
**50-54**	14.16 to 12.23	2001-2003	-5.55 (-12.69 to 2.18)	2003-2017	-0.29 (-0.65 to 0.07)					-0.96* (-1.88 to -0.04)
**55-59**	14.13 to 11.53	2001-2005	-3.90* (-7.22 to -0.46)	2005-2017	-0.33 (-0.93 to 0.28)					-1.23* (-2.11 to -0.35)
**60-64**	14.36 to 10.88	2001-2003	-6.54 (-14.97 to 2.73)	2003-2017	-1.10* (-1.50 to -0.70)					-1.79* (-2.88 to -0.70)
**65-69**	15.08 to 9.93	2001-2017	-2.44* (-2.92 to -1.96)							-2.44* (-2.92 to -1.96)
**70-74**	13.09 to 9.19	2001-2017	-2.27* (-2.70 to -1.84)							-2.27* (-2.70 to -1.84)
**75-80**	11.58 to 8.85	2001-2017	-2.07* (-2.57 to -1.56)							-2.07* (-2.57 to -1.56)
**80+**	10.70 to 6.95	2001-2012	-3.58* (-4.22 to -2.94)	2012-2017	-0.63 (-2.98 to 1.78)					-2.67* (-3.43 to -1.90)

Abbreviations: SCC, squamous cell carcinoma, ASI, age-specified incidence; APC, annual percent change; AAPC, average annual percent change

© 2022 Liao CI et al. *JAMA Network Open.*

† Trends based on incidence was analyzed using the Joinpoint Regression Program, version 4.8.0.1, allowing up to 3 joinpoints. AAPC is a summary measure of the trend over a pre-specified fixed interval. It was computed as a weighted average of the APC from the joinpoint model, with the weights equal to the length the APC interval.
* The APC or AAPC is significantly different from zero ($p < .05$).

© 2022 Liao CI et al. *JAMA Network Open.*

eTable 8. Age-Adjusted Incidences and Trends of Male HPV-Associated Cancers by Race and Ethnicity, Region, and Stage in USCS Public Use Databases from 2001 to 2017†

	AAI	Trend 1		Trend 2		Trend 3		2001-2017
	**2001 to 2017**	**Years**	**APC (95% CI)**	**Year**	**APC (95% CI)**	**Year**	**APC (95% CI)**	**AAPC (95% CI)**
**Male HPV associated cancers**	7.71 to 11.00	2001-2017	2.36* (2.13 to 2.59)					2.36* (2.13 to 2.59)
**Race**								
**NHW**	7.80 to 12.50	2001-2014	3.42* (3.13 to 3.72)	2014-2017	1.17 (-1.08 to 3.48)			3.00* (2.55 to 3.44)
**NHB**	10.09 to 9.15	2001-2017	-0.70* (-1.05 to -0.34)					-0.70* (-1.05 to -0.34)
**Hispanic**	6.42 to 6.86	2001-2017	0.46 (-0.08 to 1.00)					0.46 (-0.08 to 1.00)
**NHAPI**	2.51 to 2.92	2001-2017	0.90* (0.15 to 1.65)					0.90* (0.15 to 1.65)
**Other or Unknown**	8.43 to 20.90	2001-2017	4.57* (3.55 to 5.61)					4.57* (3.55 to 5.61)
**Region**								
**Northeast**	7.53 to 10.62	2001-2017	2.24* (2.02 to 2.47)					2.24* (2.02 to 2.47)
**Midwest**	7.06 to 11.49	2001-2017	3.00* (2.70 to 3.30)					3.00* (2.70 to 3.30)
**South**	8.61 to 11.95	2001-2017	2.22* (1.96 to 2.49)					2.22* (1.96 to 2.49)
**West**	7.10 to 9.32	2001-2017	1.99* (1.65 to 2.34)					1.99* (1.65 to 2.34)
**Stage**								
**Local**	2.16 to 2.10	2001-2017	0.13 (-0.15 to 0.40)					0.13 (-0.15 to 0.40)
**Regional**	4.29 to 7.23	2001-2017	3.38* (3.06 to 3.71)					3.38* (3.06 to 3.71)
**Distant**	0.64 to 1.06	2001-2005	24.79* (11.42 to 39.75)	2005-2015	2.00 (-0.28 to 4.34)	2015-2017	-24.25 (-42.61 to -0.01)	3.36 (-0.76 to 7.65)
**Unknown**	0.62 to 0.60	2001-2015	-1.90* (-2.98 to -0.81)	2015-2017	23.08* (1.16 to 49.76)			0.92 (-1.45 to 3.34)

© 2022 Liao CI et al. *JAMA Network Open.*

**Male Oropharyngeal SCC**	5.90 to 8.89	2001-2017	2.71* (2.48 to 2.93)					2.71* (2.48 to 2.93)
**Race**								
**NHW**	6.00 to 10.44	2001-2014	3.89* (3.59 to 4.19)	2014-2017	1.73 (-0.54 to 4.06)			3.48* (3.03 to 3.93)
**NHB**	8.14 to 6.52	2001-2017	-1.35* (-1.72 to -0.98)					-1.35* (-1.72 to -0.98)
**Hispanic**	4.32 to 4.51	2001-2017	0.62 (-0.01 to 1.26)					0.62 (-0.01 to 1.26)
**NHAPI**	1.90 to 2.18	2001-2017	1.19* (0.23 to 2.15)					1.19* (0.23 to 2.15)
**Other or Unknown**	5.87 to 16.44	2001-2017	5.48* (4.49 to 6.48)					5.48* (4.49 to 6.48)
**Region**								
**Northeast**	5.64 to 8.41	2001-2017	2.57* (2.34 to 2.79)					2.57* (2.34 to 2.79)
**Midwest**	5.50 to 9.47	2001-2017	3.41* (3.10 to 3.72)					3.41* (3.10 to 3.72)
**South**	6.70 to 9.71	2001-2017	2.47* (2.20 to 2.85(					2.47* (2.20 to 2.75)
**West**	5.26 to 7.42	2001-2017	2.48* (2.11 to 2.85)					2.48* (2.11 to 2.85)
**Stage**								
**Local**	1.12 to 1.04	2001-2005	-2.66 (-5.57 to 0.35)	2005-2017	0.51 (-0.01 to 1.04)			-0.29 (-1.05 to 0.48)
**Regional**	3.82 to 6.52	2001-2005	0.53 (-2.33 to 3.48)	2005-2017	3.97* (3.53 to 4.42)			3.10* (2.37 to 3.84)
**Distant**	0.55 to 0.90	2001-2004	37.60* (22.93 to 54.02)	2004-2015	2.51* (1.45 to 3.57)	2015-2017	-27.20* (-37.38 to -15.37)	3.79* (1.20 to 6.45)
**Unknown**	0.40 to 0.43	2001-2014	-2.43* (-4.18 to -0.12)	2014-2017	18.70 (-3.41 to 45.88)			1.23 (-2.62 to 5.22)
**Male Anal and Rectal SCC**	0.99 to 1.31	2001-2009	3.35* (2.13 to 4.59)	2009-2017	0.34 (-0.68 to 1.36)			1.83* (1.12 to 2.55)
**Race**								
**NHW**	1.02 to 1.34	2001-2017	1.85* (1.30 to 4.59)					1.85* (1.30 to 2.40)
**NHB**	1.16 to 1.82	2001-2005	11.28* (2.06 to 21.34)	2005-2017	0.90 (-0.39 to 2.22)			3.40* (1.22 to 5.63)

© 2022 Liao CI et al. *JAMA Network Open.*

**Hispanic**	0.68 to 0.94	2001-2017	0.98* (0.05 to 1.92)					0.98* (0.05 to 1.92)
**Region**								
**Northeast**	1.04 to 1.36	2001-2017	1.94* (1.23 to 2.65)					1.94* (1.23 to 2.65)
**Midwest**	0.77 to 1.22	2001-2004	9.78* (0.66 to 19.73)	2004-2017	1.56* (0.80 to 2.33)			3.05* (1.46 to 4.68)
**South**	1.03 to 1.42	2001-2005	7.20* (1.33 to 13.40)	2005-2017	1.20* (0.34 to 2.08)			2.67* (1.25 to 4.11)
**West**	1.10 to 1.19	2001-2017	0.37 (-0.38 to 1.13)					0.37 (-0.38 to 1.13)
**Stage**								
**Local**	0.53 to 0.61	2001-2009	2.35* (0.96 to 3.75)	2009-2017	-0.41 (-1.60 to 0.80)			0.96* (0.14 to 1.79)
**Regional**	0.24 to 0.46	2001-2017	3.50* (2.53 to 4.48)					3.50* (2.53 to 4.48)
**Distant**	0.06 to 0.13	2001-2004	17.65* (0.50 to 37.71)	2004-2014	4.73* (2.61 to 6.89)	2014-2017	-6.61 (-15.24 to 2.89)	4.76* (1.46 to 8.17)
**Unknown**	0.15 to 0.11	2001-2017	-1.38* (-2.03 to -0.71)					-1.38* (-2.03 to -0.71)
**Male Penile SCC**	0.82 to 0.80	2001-2017	0.13 (-0.30 to 0.56)					0.13 (-0.30 to 0.56)
**Race**								
**NHW**	0.79 to 0.72	2001-2017	0.10 (-0.48 to 0.69)					0.10 (-0.48 to 0.69)
**NHB**	0.80 to 0.80	2001-2017	-0.71 (-1.63 to 0.22)					-0.71 (-1.63 to 0.22)
**Hispanic**	1.42 to 1.40	2001-2017	-0.42 (-1.54 to 0.71)					-0.42 (-1.54 to 0.71)
**Region**								
**Northeast**	0.84 to 0.85	2001-2017	0.02 (-0.90 to 0.95)					0.02 (-0.90 to 0.95)
**Midwest**	0.80 to 0.81	2001-2017	0.31 (-0.18 to 0.80)					0.31 (-0.18 to 0.80)
**South**	0.88 to 0.82	2001-2017	0.04 (-0.42 to 0.51)					0.41 (-0.42 to 0.51)
**West**	0.74 to 0.71	2001-2017	0.22 (-0.47 to 0.92)					0.22 (-0.47 to 0.92)

© 2022 Liao CI et al. *JAMA Network Open.*

**Stage**								
**Local**	0.51 to 0.45	2001-2017	-0.53* (-1.05 to -0.01)					-0.53* (-1.05 to -0.01)
**Regional**	0.23 to 0.25	2001-2017	1.33* (0.58 to 2.10)					1.33* (0.58 to 2.10)
**Distant**	0.02 to 0.03	2001-2017	2.03* (0.54 to 3.53)					2.03* (0.54 to 3.53)
**Unknown**	0.06 to 0.06	2001-2015	-3.18* (-5.17 to -1.13)	2015-2017	41.56 (-0.83 to 102.07)			1.53 (-2.77 to 6.02)

Abbreviations: SCC, squamous cell carcinoma, AAI, age-adjusted incidence (adjusted to the 2000 US standard population); APC, annual percent change; AAPC, average annual percent change; NHW, Non-Hispanic White; NHB, Non-Hispanic Black; NHAPI, Non-Hispanic Asians or Pacific Islander; Other/Unknown (included Non-Hispanic American Indian/Alaska Native, Other unspecified, or Unknown).
† Trends based on incidence was analyzed using the Joinpoint Regression Program, version 4.8.0.1, allowing up to 3 joinpoints. AAPC is a summary measure of the trend over a pre-specified fixed interval. It was computed as a weighted average of the APC from the joinpoint model, with the weights equal to the length the APC interval.
§ Merged summary stage included local (localized only), regional (regional, direct extension only, regional lymph nodes only, direct extension and regional lymph nodes, or NOS), distant (distant site(s)/node(s) involved), and unknown (not applicable, unknown, unstaged, unspecified, or death certificate-only).
* The APC or AAPC is significantly different from zero ($p < .05$).

© 2022 Liao CI et al. *JAMA Network Open.*

eTable 9. Age-Specified Incidences and Trends of Male HPV-Associated Cancers by Age Group in USCS Public Use Databases from 2001 to 2017†

	ASI	Trend 1		Trend 2		2001-2017
**Age Group**	**2001 to 2017**	**Years**	**APC (95% CI)**	**Year**	**APC (95% CI)**	**AAPC (95% CI)**
**Male HPV associated cancer**						
**25-29**	0.22 to 0.21	2001-2017	0.20 (-1.36 to 1.78)			0.20 (-1.36 to 1.78)
**30-34**	0.65 to 0.60	2001-2017	-0.88 (-1.90 to 0.14)			-0.88 (-1.90 to 0.14)
**35-39**	2.34 to 1.90	2001-2017	-2.21* (-3.16 to -1.25)			-2.21* (-3.16 to -1.25)
**40-44**	5.54 to 5.02	2001-2007	2.81* (1.46 to 4.17)	2007-2017	-2.75* (-3.39 to -2.12)	-0.71* (-1.28 to -0.13)
**45-49**	11.40 to 12.49	2001-2013	1.30* (0.80 to 1.81)	2013-2017	-3.09* (-5.66 to -0.45)	0.18 (-0.51 to 0.88)
**50-54**	17.86 to 24.31	2001-2009	2.89* (1.90 to 3.89)	2009-2017	0.73 (-0.12 to 1.59)	1.80* (1.22 to 2.39)
**55-59**	22.14 to 34.08	2001-2009	4.18* (3.04 to 5.33)	2009-2017	1.53* (0.66 to 2.41)	2.85* (2.20 to 3.49)
**60-64**	25.78 to 41.91	2001-2017	3.43* (3.05 to 3.80)			3.43* (3.05 to 3.80)
**65-69**	26.37 to 42.57	2001-2017	3.52* (3.11 to 3.93)			3.52* (3.11 to 3.93)
**70-74**	26.47 to 41.59	2001-2017	3.35* (2.92 to 3.77)			3.35* (2.92 to 3.77)
**75-79**	22.46 to 35.79	2001-2017	3.31* (2.88 to 3.74)			3.31* (2.88 to 3.74)
**80+**	18.95 to 27.79	2001-2017	2.67* (2.32 to 3.03)			2.67* (2.32 to 3.03)
**Male Oropharyngeal SCC**						
**30-34**	0.34 to 0.30	2001-2017	-1.06 (-2.64 to 0.55)			-1.06 (-2.64 to 0.55)
**35-39**	1.08 to 1.10	2001-2017	-1.23 (-2.47 to 0.02)			-1.23 (-2.47 to 0.02)
**40-44**	3.73 to 3.77	2001-2007	2.38* (0.50 to 4.29)	2007-2017	-1.60* (-2.47 to -0.72)	-0.13 (-0.92 to 0.68)

© 2022 Liao CI et al. *JAMA Network Open.*

**45-49**	9.44 to 9.99	2001-2017	0.30 (-0.08 to 0.67)			0.30 (-0.08 to 0.67)
**50-54**	15.68 to 20.35	2001-2009	2.51* (1.50 to 3.54)	2009-2017	0.63 (-0.25 to 1.52)	1.57* (0.97 to 2.18)
**55-59**	18.66 to 29.66	2001-2008	4.75* (3.33 to 6.18)	2008-2017	1.75* (1.03 to 2.48)	3.05* (2.39 to 3.72)
**60-64**	20.73 to 36.63	2001-2017	3.83* (3.48 to 4.18)			3.83* (3.48 to 4.18)
**65-69**	20.33 to 36.48	2001-2017	4.24* (3.76 to 4.73)			4.24* (3.76 to 4.73)
**70-74**	19.89 to 33.35	2001-2017	3.91* (3.45 to 4.37)			3.91* (3.45 to 4.37)
**75-79**	15.21 to 26.88	2001-2017	3.90* (3.37 to 4.43)			3.90* (3.37 to 4.43)
**80+**	10.67 to 17.28	2001-2017	3.41* (2.92 to 3.89)			3.41* (2.92 to 3.89)
**Male Anal and Rectal SCC**						
**30-34**	0.17 to 0.23	2001-2017	-0.14 (-2.96 to 2.77)			-0.14 (-2.96 to 2.77)
**35-39**	1.01 to 0.57	2001-2017	-4.27 (-5.63 to -2.89)			-4.27 (-5.63 to -2.89)
**40-44**	1.38 to 0.90	2001-2007	5.14* (1.38 to 9.04)	2007-2017	-6.86* (-8.66 to -5.03)	-2.53* (-4.12 to -0.92)
**45-49**	1.48 to 1.87	2001-2010	6.75* (4.67 to 8.88)	2010-2017	-5.47* (-8.06 to -2.80)	1.22 (-0.27 to 2.73)
**50-54**	1.52 to 3.00	2001-2009	7.20* (4.14 to 10.35)	2009-2017	0.93 (-1.39 to 3.31)	4.02* (2.29 to 5.77)
**55-59**	2.27 to 3.26	2001-2017	3.30* (2.32 to 4.28)			3.30* (2.32 to 4.28)
**60-64**	2.63 to 3.69	2001-2017	2.79* (1.90 to 3.68)			2.79* (1.90 to 3.68)
**65-69**	2.85 to 3.83	2001-2017	1.71* (0.90 to 2.52)			1.71* (0.90 to 2.52)
**70-74**	3.10 to 4.78	2001-2015	1.30* (0.39 to 2.22)	2015-2017	13.28 (-2.26 to 31.29)	2.73* (0.89 to 4.60)
**75-79**	2.72 to 4.49	2001-2017	2.75* (1.91 to 3.59)			2.75* (1.91 to 3.59)
**80+**	2.73 to 4.09	2001-2017	2.89* (1.66 to 4.14)			2.89* (1.66 to 4.14)

© 2022 Liao CI et al. *JAMA Network Open.*

**Male Penile SCC**						
**40-44**	0.44 to 0.34	2001-2017	-0.58 (-1.78 to 0.64)			-0.58 (-1.78 to 0.64)
**45-49**	0.49 to 0.63	2001-2017	1.64* (0.21 to 3.09)			1.64* (0.21 to 3.09)
**50-54**	0.66 to 0.95	2001-2017	0.59 (-0.64 to 1.83)			0.59 (-0.64 to 1.83)
**55-59**	1.21 to 1.16	2001-2017	-1.05* (-1.82 to -0.28)			-1.05* (-1.82 to -0.28)
**60-64**	2.42 to 1.58	2001-2017	-1.59* (-2.48 to -0.68)			-1.59* (-2.48 to -0.68)
**65-69**	3.19 to 2.26	2001-2017	-1.68* (-2.31 to -1.04)			-1.68* (-2.31 to -1.04)
**70-74**	3.48 to 3.45	2001-2017	0.65 (-0.39 to 1.70)			0.65 (-0.39 to 1.70)
**75-79**	4.52 to 4.42	2001-2017	1.13* (0.45 to 1.82)			1.13* (0.45 to 1.82)
**80+**	5.55 to 6.42	2001-2017	0.86* (0.02 to 1.72)			0.86* (0.02 to 1.72)

Abbreviations: SCC, squamous cell carcinoma, ASI, age-specified incidence; APC, annual percent change; AAPC, average annual percent change

† Trends based on incidence was analyzed using the Joinpoint Regression Program, version 4.8.0.1, allowing up to 3 joinpoints. AAPC is a summary measure of the trend over a pre-specified fixed interval. It was computed as a weighted average of the APC from the joinpoint model, with the weights equal to the length the APC interval.

* The APC or AAPC is significantly different from zero ($p < .05$).

© 2022 Liao CI et al. *JAMA Network Open.*

eTable 10. Age-Specified Incidences and Trends of Oropharyngeal SCC by Sex and Race and Ethnicity in USCS Public Use Databases from 2001 to 2017†

		United States			NHW			NHB			Hispanic	
**Male**		**ASI**	**2001-2017**		**ASI**	**2001-2017**		**ASI**	**2001-2017**		**ASI**	**2001-2017**
**Age Group**	**N**	**2001 to 2017**	**AAPC (95% CI)**	**N**	**2001 to 2017**	**AAPC (95% CI)**	N	**2001 to 2017**	**AAPC (95% CI)**	**N**	**2001 to 2017**	**AAPC (95% CI)**
**30-34**	520	0.34 to 0.30	-1.06 (-2.64 to 0.55)	-	-	-	-	-	-	-	-	-
**35-39**	2019	1.08 to 1.10	-1.23 (-2.47 to 0.02)	1525	1.26 to 1.34	-0.48 (-1.80 to 0.87)	-	-	-	-	-	-
**40-44**	7338	3.73 to 3.77	-0.13 (-0.92 to 0.68)	5940	4.17 to 4.92	0.86* (0.17 to 1.55)	759	3.54 to 3.18	-1.60 (-3.22 to 0.05)	454	1.82 to 1.61	-0.71 (-2.28 to 0.90)
**45-49**	18921	9.44 to 9.99	0.30 (-0.08 to 0.67)	15629	9.94 to 13.06	1.32* (0.47 to 2.18)	1871	12.47 to 7.38	-3.83* (-5.06 to -2.59)	1005	5.02 to 3.34	-1.67* (-3.12 to -0.21)
**50-54**	33050	15.68 to 20.35	1.57* (0.97 to 2.18)	27661	15.93 to 25.76	2.96* (2.38 to 3.55)	3187	22.31 to 12.90	-3.39* (-4.29 to -2.48)	1558	10.87 to 7.82	-1.44* (-2.44 to -0.43)
**55-59**	41228	18.66 to 29.66	3.05* (2.39 to 3.72)	34677	18.72 to 35.29	4.10* (3.37 to 4.83)	3853	28.83 to 20.46	-2.38* (-2.85 to -1.91)	1868	11.92 to 13.18	0.68 (-0.47 to 1.84)
**60-64**	38371	20.73 to 36.63	3.83* (3.48 to 4.18)	32383	20.86 to 42.87	4.59* (3.92 to 5.27)	3468	28.88 to 23.70	-0.73 (-1.71 to 0.26)	1752	15.54 to 15.56	0.56 (-0.48 to 1.61)
**65-69**	29704	20.33 to 36.48	4.24* (3.76 to 4.73)	25091	20.30 to 41.58	5.13* (4.67 to 5.60)	2630	31.72 to 24.08	-0.58 (-1.65 to 0.51)	1389	13.55 to 18.26	1.27 (-0.17 to 2.73)
**70-74**	19207	19.89 to 33.35	3.91* (3.45 to 4.37)	16158	19.42 to 36.85	4.63* (4.15 to 5.11)	1669	33.37 to 26.20	-0.18 (-1.12 to 0.76)	970	17.48 to 19.22	1.27 (-0.16 to 2.72)
**75-80**	11610	15.21 to 26.88	3.90* (3.37 to 4.43)	9886	15.17 to 28.65	4.45* (3.80 to 5.11)	880	15.45 to 23.84	1.02 (-0.53 to 2.59)	569	17.82 to 20.21	0.89 (-4.97 to 7.12)
**80+**	9201	10.67 to 17.28	3.41* (2.92 to 3.89)	7871	10.69 to 17.90	3.90* (3.37 to 4.43)	629	10.73 to 17.59	0.79 (-1.39 to 3.01)	-	-	-
		**United States**			**NHW**			**NHB**			**Hispanic**	
**Female**		**ASI**	**2001-2017**		**ASI**	**2001-2017**		**ASI**	**2001-2017**		**ASI**	**2001-2017**
**Age Group**	**N**	**2001-2017**	**AAPC (95% CI)**	**N**	**2001-2017**	**AAPC (95% CI)**	**N**	**2001-2017**	**AAPC (95% CI)**	**N**	**2001-2017**	**AAPC (95% CI)**
**35-39**	696	0.34 to 0.44	1.03 (-1.14 to 3.25)	537	0.44 to 0.61	2.14 (-0.45 to 4.80)	-	-	-	-	-	-

© 2022 Liao CI et al. *JAMA Network Open.*

**40-44**	1675	1.03 to 0.60	-1.63* (-3.05 to -0.19)	1244	0.97 to 0.78	0.06 (-1.44 to 1.58)	-	-	-	-	-	-
**45-49**	3790	1.75 to 1.78	0.78 (-1.33 to 2.94)	2902	1.75 to 2.35	1.17* (0.16 to 2.20)	-	-	-	-	-	-
**50-54**	6448	3.02 to 3.38	1.08 (-0.47 to 2.66)	5110	3.16 to 4.12	2.03* (0.36 to 3.73)	864	4.14 to 2.87	-0.89 (-2.68 to 0.93)	-	-	-
**55-59**	7984	4.20 to 5.39	2.35* (1.76 to 2.94)	6454	4.51 to 6.16	3.06* (2.42 to 3.70)	983	5.21 to 4.86	-0.78 (-2..10 to 0.56)	-	-	-
**60-64**	7887	5.02 to 6.38	0.92* (0.35 to 1.49)	6565	5.15 to 7.34	1.40* (0.79 to 2.01)	819	7.23 to 5.26	-1.41 (-2.96 to 0.17)	-	-	-
**65-69**	7193	5.57 to 6.36	0.54 (-0.15 to 1.23)	6112	5.64 to 7.24	0.99* (0.19 to 1.81)	639	6.69 to 4.61	-2.09* (-3.46 to -0.70)	-	-	-
**70-74**	5848	7.04 to 6.30	0.10 (-0.47 to 0.67)	5040	7.41 to 6.99	0.48 (-0.19 to 1.15)	466	6.31 to 4.95	-1.98* (-3.45 to -0.48)	-	-	-
**75-80**	4408	6.46 to 6.06	0.40 (-0.36 to 1.17)	3829	6.73 to 6.81	1.00* (0.10 to 1.92)	-	-	-	-	-	-
**80+**	4993	4.31 to 4.30	0.94* (0.22 to 1.66)	4397	4.46 to 4.64	1.25* (0.55 to 1.96)	-	-	-	-	-	-

Abbreviations: SCC, squamous cell carcinoma, ASI, age-specified incidence; APC, annual percent change; AAPC, average annual percent change; NHW, Non-Hispanic White; NHB, Non-Hispanic Black; N, case number

† Trends based on incidence was analyzed using the Joinpoint Regression Program, version 4.8.0.1, allowing up to 3 joinpoints. AAPC is a summary measure of the trend over a pre-specified fixed interval. It was computed as a weighted average of the APC from the joinpoint model, with the weights equal to the length the APC interval. When case number was less than 16, SEER*Stat would hide statistic result and Joinpoint Regression Program could not analyze the incidences or trends.

* The APC or AAPC is significantly different from zero (*p* < .05).

© 2022 Liao CI et al. *JAMA Network Open.*

eTable 11. Cervical Cancer Screening Trends in BRFSS (Ever Received any Cervical Cancer Screening)

**BRFSS**	**Yes (%)**		**Trend 1**			**Trend 2**			**2001-2016**
**Age Group (Female)**	**2001 to 2016**		**Years**	**APC (95% CI)**		**Year**	**APC (95% CI)**		**AAPC (95% CI)**
**18-24**	87.0 to 51.4		2001-2011	-2.20* (-2.76 to -1.63)		2011-2016	-6.36* (-7.91 to -4.79)		-3.61* (-4.19 to -3.02)
**25-29**	96.2 to 90.6		2001-2009	-0.18 (-0.41 to 0.06)		2009-2016	-0.76* (-1.05 to -0.48)		-0.45* (-0.61 to -0.29)
**30-34**	97.2 to 94.5		2001-2016	-0.21* (-0.30 to -0.13)		-	-		-0.21* (-0.30 to -0.13)

© 2022 Liao CI et al. *JAMA Network Open.*

eFigure. Average Annual Percent Change in Oropharyngeal SCC by Sex in USCS Public Use Databases from 2001 to 2017

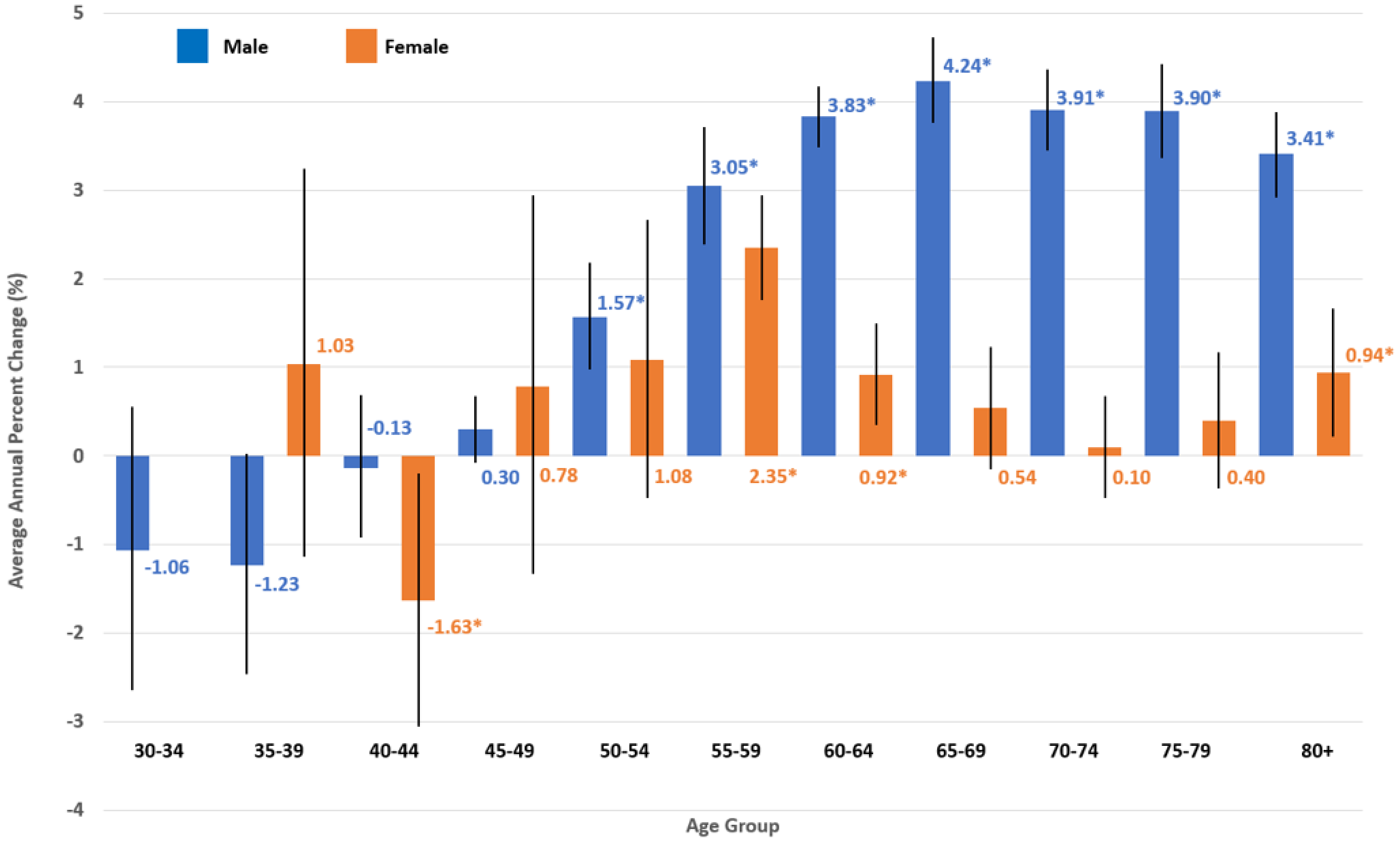

Abbreviations: SCC, squamous cell carcinoma; APC, annual percent change; AAPC, average annual percent change.
Trends based on incidence was analyzed using the Joinpoint Regression Program, version 4.8.0.1, allowing up to 3 joinpoints. AAPC is a summary measure of the trend over a pre-specified fixed interval. It was computed as a weighted average of the APC from the joinpoint model, with the weights equal to the length the APC interval. Vertical lines indicate the associated 95% confidence intervals.
* The APC or AAPC is significantly different from zero ($p < .05$).

© 2022 Liao CI et al. *JAMA Network Open.*
